# Pipeline for illumination correction of images for high-throughput microscopy

**DOI:** 10.1111/jmi.12178

**Published:** 2014-09-16

**Authors:** S Singh, M-A Bray, TR Jones, AE Carpenter

**Affiliations:** Broad Institute of MIT and HarvardCambridge, Massachusetts, U.S.A.

**Keywords:** Fluorescence microscopy, high-throughput microscopy, illumination correction, shading correction, vignetting

## Abstract

The presence of systematic noise in images in high-throughput microscopy experiments can significantly impact the accuracy of downstream results. Among the most common sources of systematic noise is non-homogeneous illumination across the image field. This often adds an unacceptable level of noise, obscures true quantitative differences and precludes biological experiments that rely on accurate fluorescence intensity measurements.

In this paper, we seek to quantify the improvement in the quality of high-content screen readouts due to software-based illumination correction. We present a straightforward illumination correction pipeline that has been used by our group across many experiments. We test the pipeline on real-world high-throughput image sets and evaluate the performance of the pipeline at two levels: (a) Z′-factor to evaluate the effect of the image correction on a univariate readout, representative of a typical high-content screen, and (b) classification accuracy on phenotypic signatures derived from the images, representative of an experiment involving more complex data mining. We find that applying the proposed post-hoc correction method improves performance in both experiments, even when illumination correction has already been applied using software associated with the instrument.

To facilitate the ready application and future development of illumination correction methods, we have made our complete test data sets as well as open-source image analysis pipelines publicly available. This software-based solution has the potential to improve outcomes for a wide-variety of image-based HTS experiments.

## Introduction

Automated microscopes have become widely used, allowing acquisition of thousands of images at rates previously unattainable. Image processing software allows the automatic, quantitative analysis of these images. Uneven illumination of the field of view is often tolerable if images are analyzed qualitatively – that is, viewed by an expert. However, when precise quantitative measurements are needed, variation in illumination can contribute a level of noise that confounds an experiment's goals.

Several approaches can mitigate this problem, also known as intensity nonuniformity, uneven shading or vignetting. Improvements to the optical path can help, such as using a light source as uniform as possible (e.g. using fibre optics) and reducing aberrations in the optical path such as dust or nonuniform filters (due to manufacturing conditions or burn-in). Here, we focus on software approaches, which can further reduce intensity anomalies and improve data quality in high-throughput microscopy experiments.

### White-referencing approaches to illumination correction are insufficient for quantitative high-throughput microscopy

For brightfield images, dividing each image by an image of a blank field of view taken immediately after each exposure, and then normalizing the resulting image provides simple correction but is not robust against artefacts (e.g. dust) or changes in overall brightness of the image (Schultz *et al.*, 1974; Regitnig *et al.*, 2003; Piccinini *et al.*, 2012). In addition, this is not practical for high-throughput experiments or for fluorescence images where a different sample must be used to collect the white reference image. For fluorescence microscopy, a comparable method called ‘white referencing’, ‘flat field correction’ or ‘shading correction’ can be used, and is included with many commercial microscopes’ software (Model & Burkhardt, 2001; Zwier *et al.*, 2004). This requires collecting a white reference image of a uniformly fluorescent sample (e.g. free fluorescent dye), which is then divided or subtracted from each collected image, often followed by a normalization step (reviewed in Likar *et al.*, 2000 and Piccinini *et al.*, 2012). Further improvement of the image may result by preceding this correction by subtracting a dark-current image acquired with the shutter closed to remove the electronic noise resulting from the camera (Likar *et al.*, 2000).

There are several problems with white-referencing-based approaches to illumination correction, at least in practice. The methods require that the user properly create the appropriate white referencing images and that conditions do not change between the acquisition of the images and the collection of the experimental images. Some software only allow one white reference image to be used to correct all wavelengths, despite substantial differences in their optical paths and spectral characteristics. For white referencing to work well, images ought to be collected across a range of exposure times so that a linear curve can be fit to each pixel and applied appropriately to the actual exposure time of a given image (Hiraoka *et al*., 1987); however, this capability is not available in most microscope-associated software. Further, white referencing does not take into account the effect of the fluorescent dye being in a different chemical environment in real samples compared to a uniformly fluorescent control. In fact, often a different fluorophore which has a similar but not identical spectral range is used. It is also common for microscope users to be unfamiliar with best practices and to inappropriately apply white reference images, such as outdated images that are stored in memory with the instrument, despite changing optical conditions.

Thus, in practice, images acquired from standard or automated microscopes, even with white referencing, are generally adequate for visual inspection but unsatisfactory for quantitative image analysis, because the intensity of an object is rendered dependent on its position within the field of view. For example, in careful studies, corrections using a white reference image of a uniformly fluorescent dye reduced the variation in mean intensity of a cell's signal from ∼20% to ∼5% (Pajor & Honeyman, 1995) and from ∼30% to ∼5% (Model & Burkhardt, 2001). In practical use, when conditions are not so carefully controlled, differences can be more substantial; in our experience, illumination can routinely vary 10–30% across a single image when using standard microscope hardware, even when white-referencing has been applied (see below).

### Retrospective approaches to illumination correction

There is therefore a strong need for illumination correction that depends on nothing but the actual images acquired during an experiment; such an approach is called data-driven, or retrospective, because it can be carried out after image acquisition and does not depend on proper white-referencing images to be taken. A retrospective approach can help overcome illumination anomalies which arise due to the absence of or incorrect application of white referencing, or due to the residual anomalies that remain even after appropriate white referencing.

As a simple example of a retrospective approach, the background variation in each image can be independently corrected by subtracting a smoothed version of the raw image. However, this relies upon certain assumptions that are rarely appropriate for all images in a high-throughput experiment, for example, that the distribution of material in each image is roughly uniform across the field of view. Retrospective methods that estimate an illumination correction function (ICF) by combining information across multiple images are more robust and thus more desirable.

(Likar *et al.* 2000) propose a retrospective method that uses a linear model of image formation consisting of additive and multiplicative shading components. The components, which are modelled as second order polynomials, are estimated by minimizing the entropy of the corrected image. With a focus on correcting intensity heterogeneity in 3D confocal images (Lee & Bajcsy, 2006) propose a method that minimizes the variation in intensity across the image, while maximizing the contrast and minimizing resulting distortions near the edges of objects. Poon *et al.* (2008) propose a retrospective method to adjust for local variation in background, where morphological filters are used to estimate intensity in the adjoining pixels near the cells. However, to estimate the global variation in background, they propose a prospective method that requires using fluorescent calibration beads *a priori*. Piccinini *et al.* (2012) recently proposed a method to correct for vignetting in bright-field images. The technique is based on the assumption that the image background is more homogenous relative to foreground, and estimates a correction function that is estimated over the background regions of the image. Other retrospective methods of illumination correction have been reviewed (Likar *et al.*, 2000; Russ, 2002; Leong *et al.*, 2003; Lee & Bajcsy, 2006; Piccinini *et al.*, 2012), but to our knowledge none have been assessed in the context of high-throughput fluorescence imaging experiments, complete with freely usable pipelines and test image sets.

### Computing illumination correction functions and assessing anomalies

**Figure 1:**
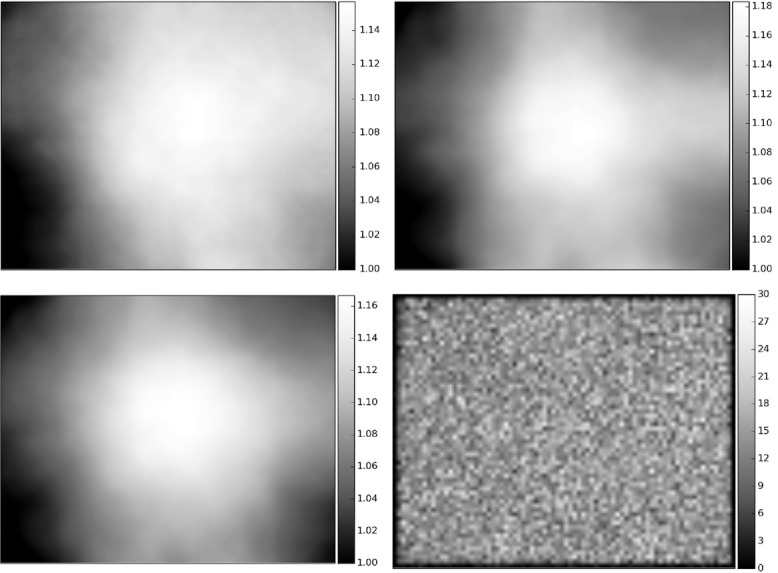
Estimating illumination correction functions. Sample ICFs for DAPI (top-left), actin (top-right) and tubulin (bottom-left). The image dimensions are 1280 × 1024 pixels. To generate an ICF, the mean image across all images in a plate is smoothed using a median filter with window size = 500 pixels. The value of this parameter was chosen manually as follows. Start with filter dimensions approximately 25% that of the image, then increase the dimensions in increments of about 10% of the image size until the ICFs appear smooth overall; the presence of bright ‘blotches’ in the ICF indicates that the local cellular intensities still dominate the global illumination pattern or that artefacts are present. The density of cells across the field-of-view is uniform (bottom-right). This check is important to confirm that there are no systematic uneven cell distribution patterns that could lead to erroneous ICFs.

We have found that a straightforward approach to retrospective illumination correction (Jones *et al.*, 2006) works well in practice for high-throughput microscopy experiments; here we validate its use. The approach is as follows. The ICF is calculated by averaging all images in an experimental batch (usually, all images for a particular channel from a particular multi-well plate), followed by smoothing using a median filter. Then, each image is corrected by dividing it by the ICF. For the results presented in this paper, we have used a median filter with window size = 500 pixels for the smoothing (Fig.1).

We have implemented the approach in freely available open-source software, CellProfiler (Carpenter *et al.*, 2006; Kamentsky *et al.*, 2011) so that it can be readily and routinely applied to large numbers of images from high-throughput microscopy experiments.

For testing, we selected a high-throughput image set BBBC021v1 (Caie *et al.*, 2010) from the Broad Bioimage Benchmark Collection (Ljosa *et al.*, 2012) that is publicly available. Images in this set have already been corrected by white referencing using the image acquisition software (MetaMorph software; images acquired on ImageXpress 5000A microscope manufactured by Molecular Devices, Union City, CA, USA). We computed ICFs using images from each 96-well plate as the experimental batch (Fig.1). We find that despite the previously applied white referencing correction, the intensity response across the field of view for these images still varies 10–30%, depending on the channel and the plate (Figs. S1 and S2 shows ICFs from all the plates in the experiment).

We have observed that for microtiter plate-based imaging experiments, ICFs vary significantly across plates, and we thus typically compute ICFs by plate-wise grouping of images. Other groupings of images, such as by row, column or site, are feasible but in our experience, including this data set, there is less variation requiring correction across row, column and site than across plates (Fig. S3, with ICFs corresponding to these groupings shown in Figs. S4–S6).

We find that viewing the functions themselves is good practice to identify quality control issues (Bray & Carpenter, 2013). For example, a sudden change in the pattern of ICFs for some plates may alert to a problem with microscope hardware, or a single unusual ICF from a plate may identify a single image with intense debris (Fig. S7).

### Influence of illumination correction on assay quality: screens using a univariate readout

The most common application of high-throughput microscopy involves selecting a single readout of interest, rank-ordering each sample with respect to measurements of that readout and selecting ‘hits’ (Singh *et al.*, 2014). We therefore tested whether our standard illumination correction method improves assay quality for an assay with a single readout. Using the BBBC021 image set, we selected wells treated with DMSO as negative controls, wells treated with Taxol as positive controls, and measured the ability to distinguish them, with and without our retrospective illumination correction method. We used the Z′-factor (Zhang *et al.*, 1999) as a measure of assay quality, but modified it to be ‘one-tailed’ (Supporting Information and Bray & Carpenter, 2013) to account for the typically asymmetric distributions of image-based readouts.

Although the BBBC021 experiment was not specifically designed as an assay for Taxol's effects, we examined images and noted that the intensity of tubulin staining distinctly increases when cells are treated with Taxol, compared to DMSO; there is prior evidence for this effect (Sum *et al.*, 2014). We thus chose this simple intensity metric as the readout for our illumination correction tests (vs. more complex readouts that are more heavily influenced by other factors such as segmentation quality). For each well, we measured the total intensity of tubulin within each cell, and computed the median of this value across all cells in the well to obtain the readout for the well. The quality of this readout is not sufficiently high to justify a real screen: without illumination correction, the Z′-factor is −0.57. This measurement was a good choice for our purposes, however, because we wanted an intensity-based readout whose baseline quality was not so high so as to leave no room for improvement upon illumination correction. Indeed, the Z′-factor improves after applying illumination correction, increasing to −0.40 (corresponding results for row, column and site-wise grouping are shown in Fig. S9). This result is consistent: we also observed that the Z′-factor improves for nearly all of the other 41 Tubulin intensity-related features (Fig. S10a; the corresponding results for row, column and site-wise grouping are shown in Figs. 10b–d). In addition, we observe that the statistical significance in the difference between means increases as well (Fig. S11).

In considering the significance of these improvements, it is important to note that these images have already been corrected for illumination using white referencing at the time of acquisition. For most experimenters, therefore, it is worth applying a simple post-hoc computational approach in order to see Z′-factor improvements of >0.10 for images that were already thought to be unaffected by illumination variation.

### Influence of illumination correction on assay quality: experiments involving multivariate profiling

Data quality is an even more serious consideration in experiments involving multivariate profiling. In profiling experiments, hundreds of measurements of cells are used simultaneously; the effects of small anomalies can be amplified and hamper sensitivity. We recently published a comparison of methods for image-based profiling and released complete ground-truth and test data sets, as well as open-source implementations of the various methods in a common software framework (Ljosa *et al.*, 2013). Here, we used these images (also from BBBC021) and methods to test whether our standard illumination correction can improve the ability to identify similarities and differences among samples of cells treated with various small molecules, in order to predict their mechanisms of action.

The image and data analysis methods to perform this task are described in detail in Ljosa *et al.* (2013); a brief summary follows. Features are extracted from each cell in an image, which are then normalized using a reference distribution defined by control cells. The image-based profile is given by computing the mean for each feature across all the cells treated with that compound. Nearest neighbour classification is used to identify the compound's mechanism of action.

**Figure 2:**
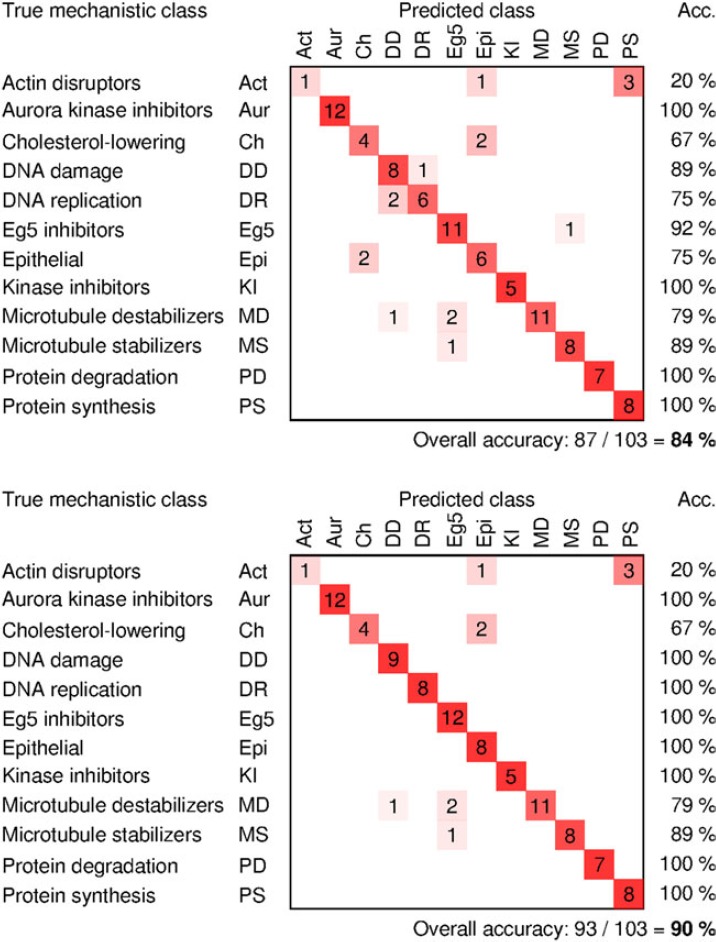
Influence of illumination correction on image-based profiling. Illumination correction improves accuracy of mechanism-of-action classification of compounds by 6% even though these images have already been white-referenced using the microscope's software. Confusion matrices show classification accuracy without the proposed method of correction (top) and with correction (bottom). ICFs were computed by plate-wise grouping of images.

The classification accuracy using these images, which have already been corrected using white-referencing, is 84%. Applying our retrospective illumination correction method increases the classification accuracy by 6% (Fig.2; the corresponding results for row, column and site-wise grouping are shown in Fig. S12). Again, application of this simple approach is worthwhile to improve data quality.

## Conclusion

To facilitate the practical application of this approach, we have implemented the necessary tools as modules in our freely available, open-source software CellProfiler for high-throughput image analysis (Carpenter *et al.*, 2006; Kamentsky *et al.*, 2011; http://www.cellprofiler.org). Example pipelines are available (Supporting Information; http://cellprofiler.org/published_pipelines.shtml), as is the test data set used in this paper (Ljosa *et al.*, 2012; http://www.broadinstitute.org/bbbc/). We have provided source code to fully reproduce the key results presented (Supporting Information).

## Supporting Information

Disclaimer: Supplementary materials have been peer-reviewed but not copyedited.

Figure S1 Variation of ICF values. **Figure S2**. Illumination correction functions based on plate-wise grouping. **Figure S3**. Grouping of images to compute ICF. **Figure S4**. Illumination correction functions based on row-wise grouping. **Figure S5**. Illumination correction functions based on column-wise grouping. **Figure S6**. Illumination correction functions based on site-wise grouping. **Figure S7**. Using ICFs for quality control. **Figure S8**. Influence of illumination correction on segmentation quality. **Figure S9**. Improvements in Z′-factor after illumination correction for a single readout. **Figure S10.(a)–(d)** Influence of illumination correction on assay quality – multiple features. **Figure S11**. Improvements in separation between means after illumination correction for a single readout. **Figure S12**. Influence of illumination correction on image-based profiling.

Appendix Source Code & Definitions.
