# Supplementary material for: Pipeline for illumination correction of images for high-throughput microscopy
Source: J Microsc. 2014 Sep 16;256(3):231–6. doi: 10.1111/jmi.12178 (PMC4359755; doi:10.1111/jmi.12178)
Supplement: Figure S1 — Variation of ICF values. Figure S2. Illumination correction functions based on plate-wise grouping. Figure S3. Grouping of images to compute ICF. Figure S4. Illumination correction functions based on row-wise grouping. Figure S5. Illumination correction functions based on column-wise grouping. Figure S6. Illumination correction functions based on site-wise grouping. Figure S7. Using ICFs for quality control. Figure S8. Influence of illumination correction on segmentation quality. Figure S9. Improvements in Z′-factor after illumination correction for a single readout. Figure S10.(a)–(d) Influence of illumination correction on assay quality – multiple features. Figure S11. Improvements in separation between means after illumination correction for a single readout. Figure S12. Influence of illumination correction on image-based profiling. [file jmi0256-0231-SD1.pdf]

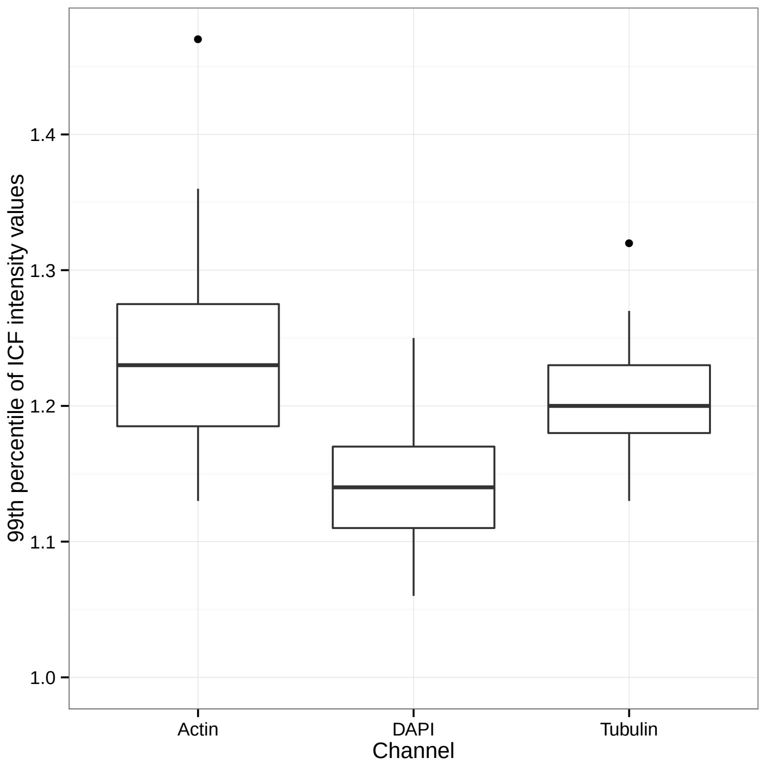

Figure S1: **Variation of ICF values.** ICFs are computed plate-wise and for each channel, the 99th percentile of the ICF values are plotted. In this experiment, the median of this value is ˜14% for the DAPI channel and ˜23% for the actin channel, despite the images having been white-referenced during acquisition.

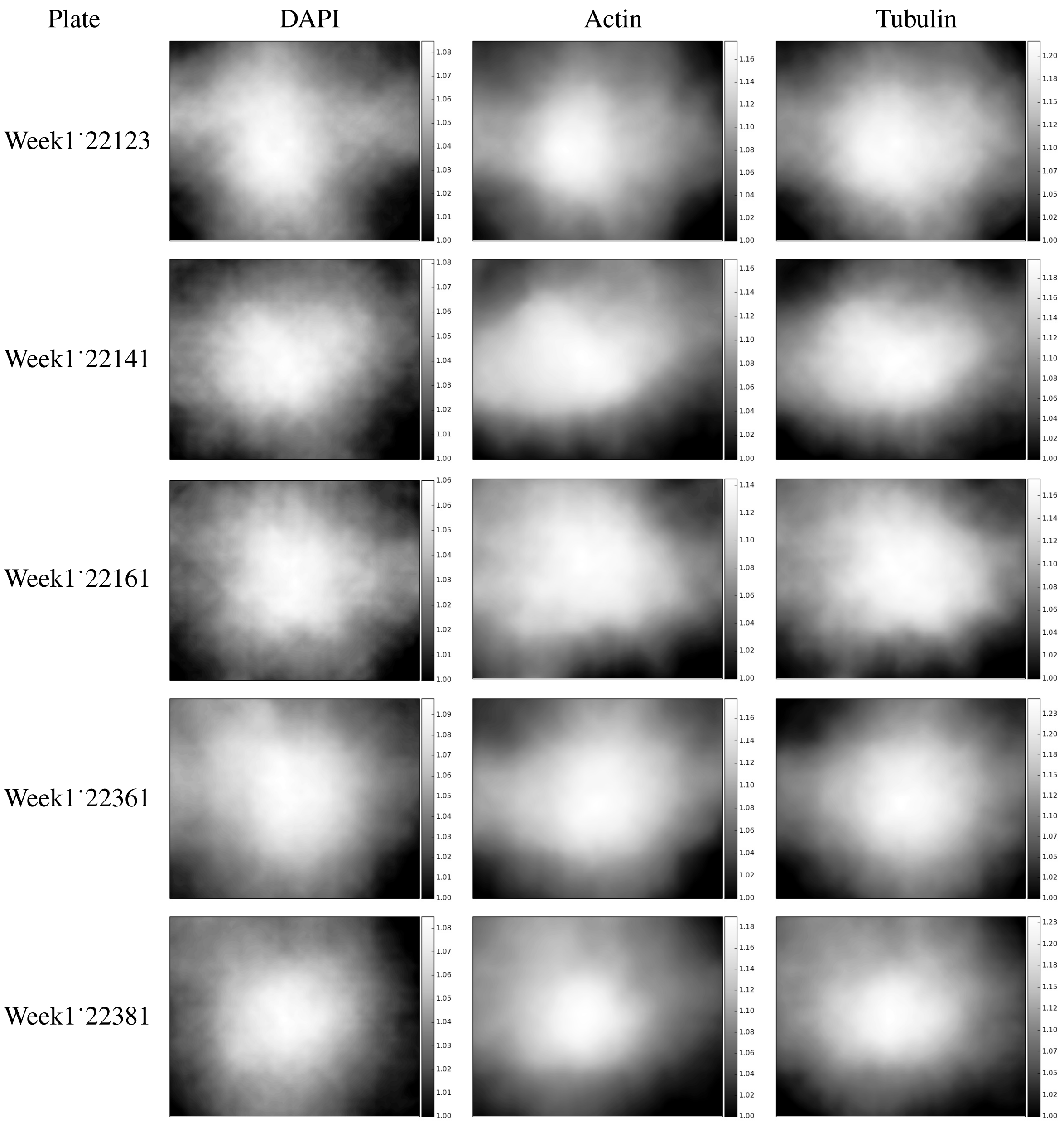

Figure S2: **Illumination correction functions based on plate-wise grouping (Plates 1-5).**

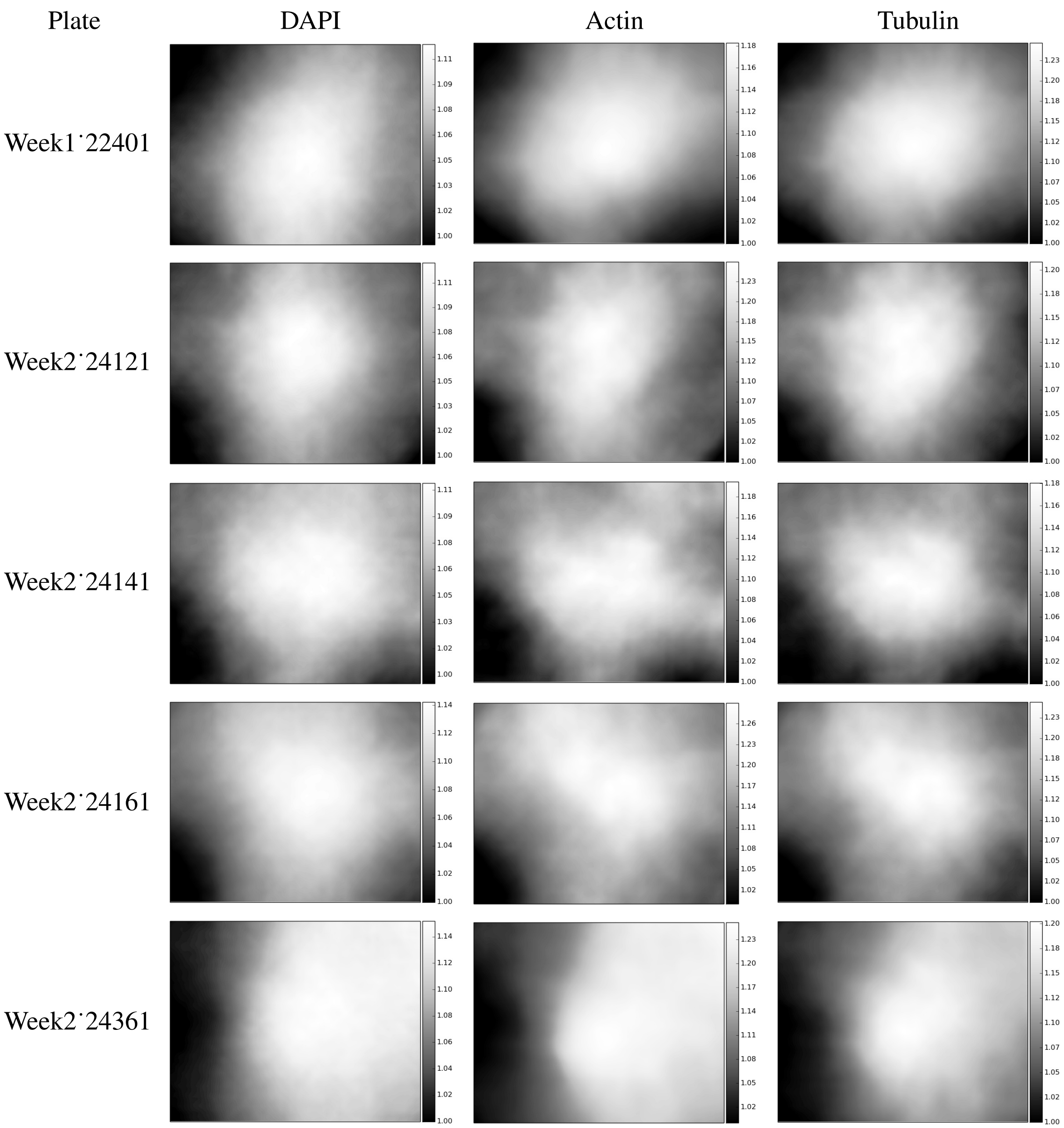

Figure S2: **Illumination correction functions based on plate-wise grouping (Plates 6-10).**

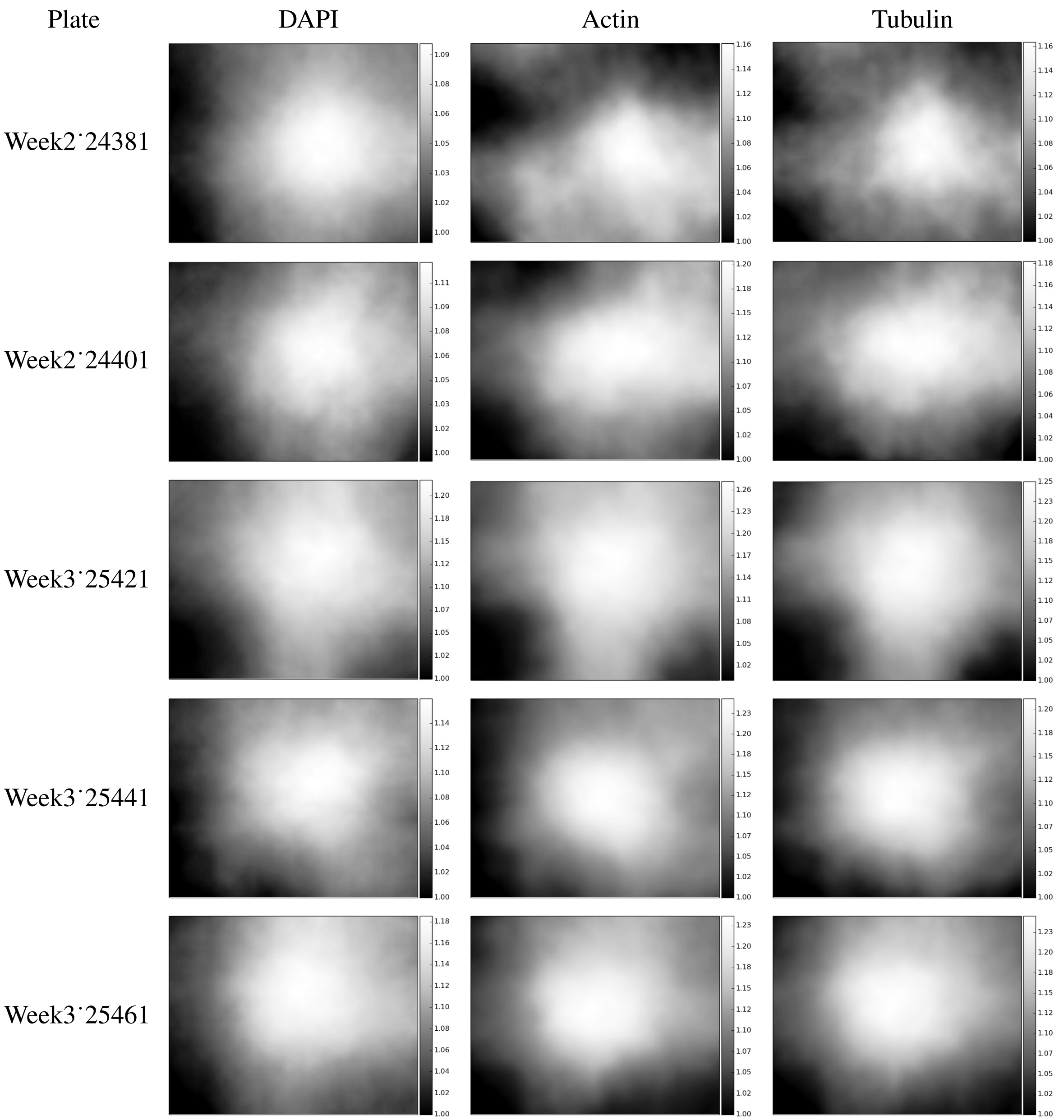

Figure S2: **Illumination correction functions based on plate-wise grouping (Plates 11-15).**

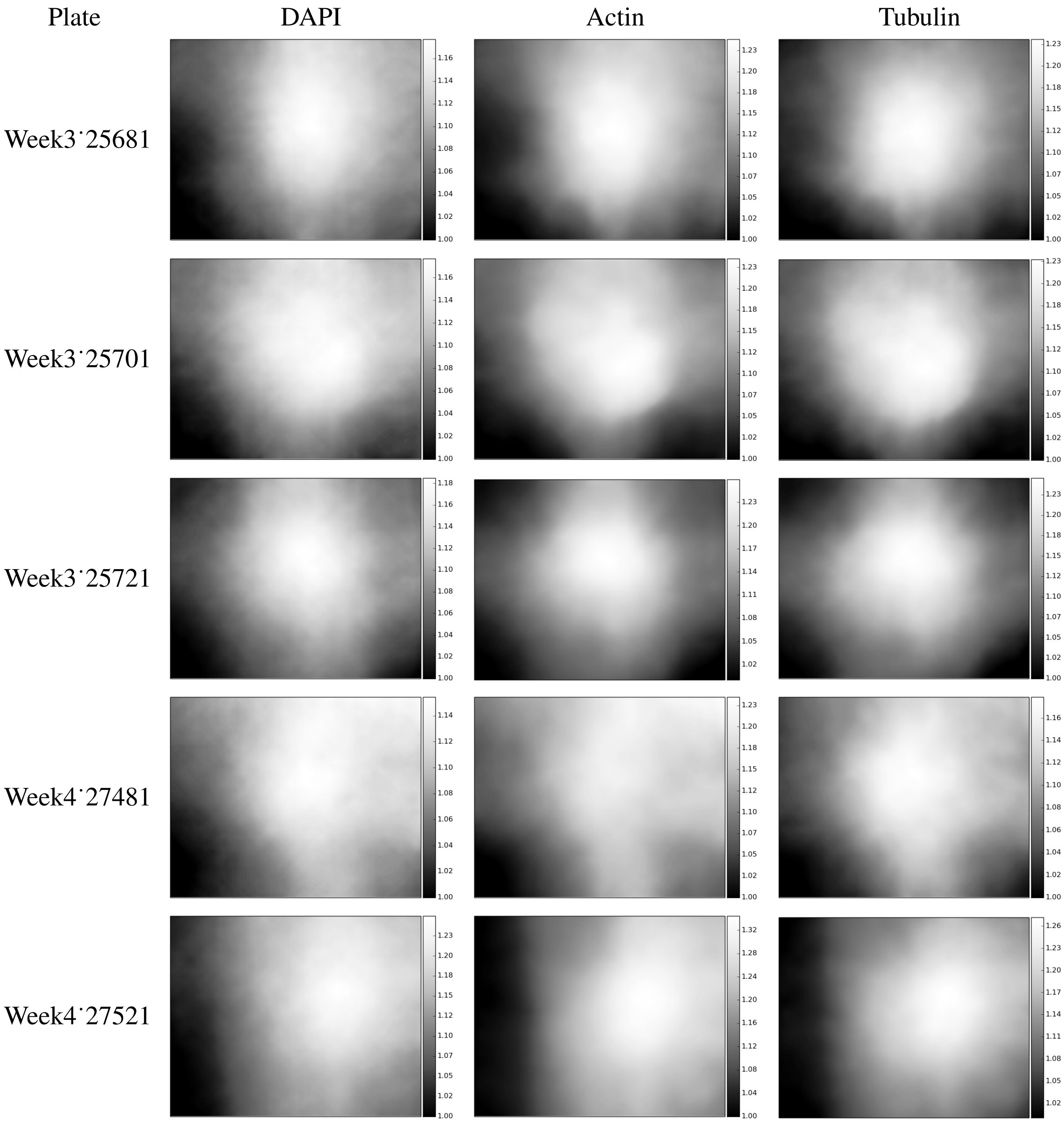

Figure S2: **Illumination correction functions based on plate-wise grouping (Plates 16-20).**

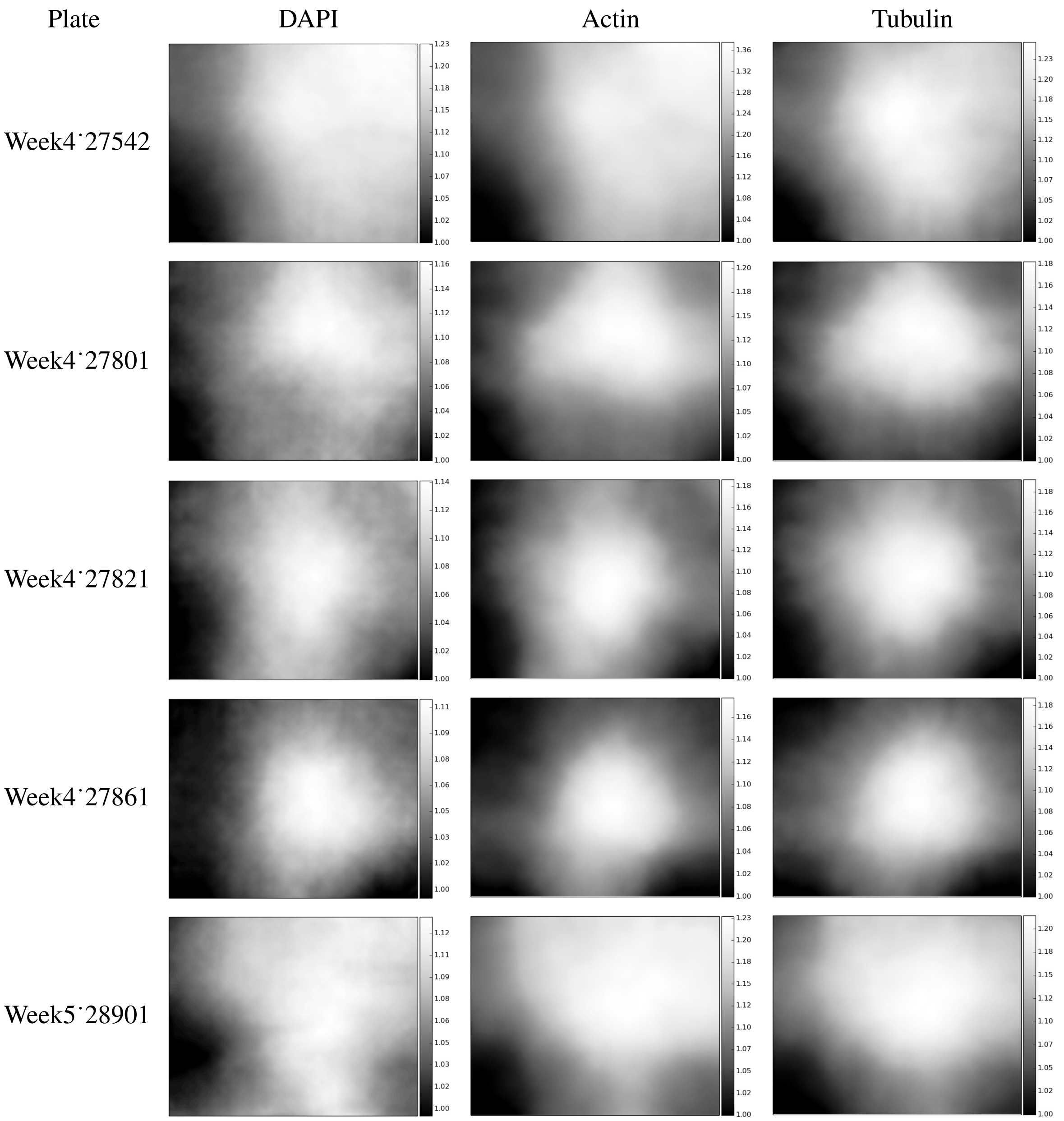

Figure S2: **Illumination correction functions based on plate-wise grouping (Plates 21-25).**

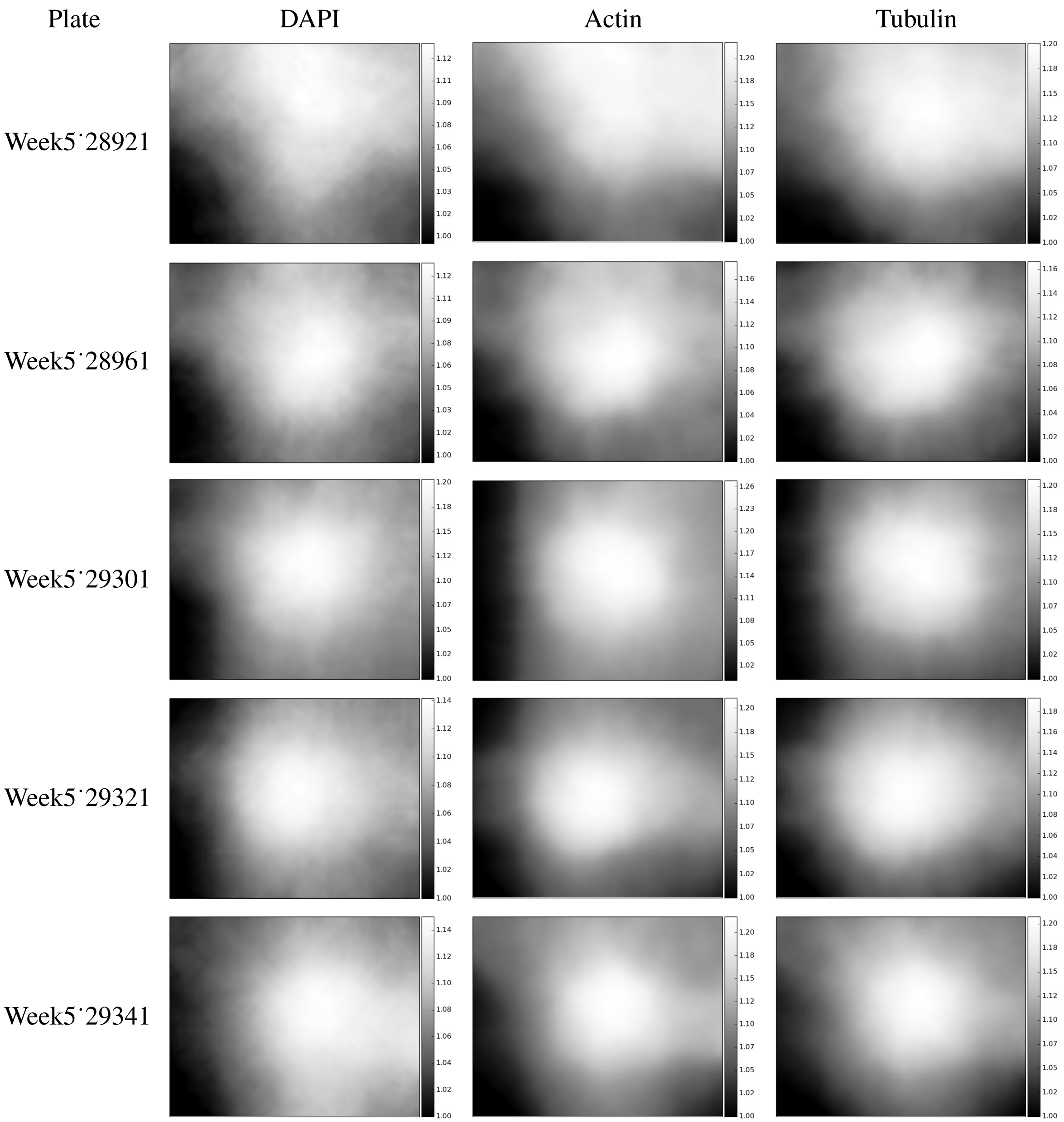

Figure S2: **Illumination correction functions based on plate-wise grouping (Plates 26-30).**

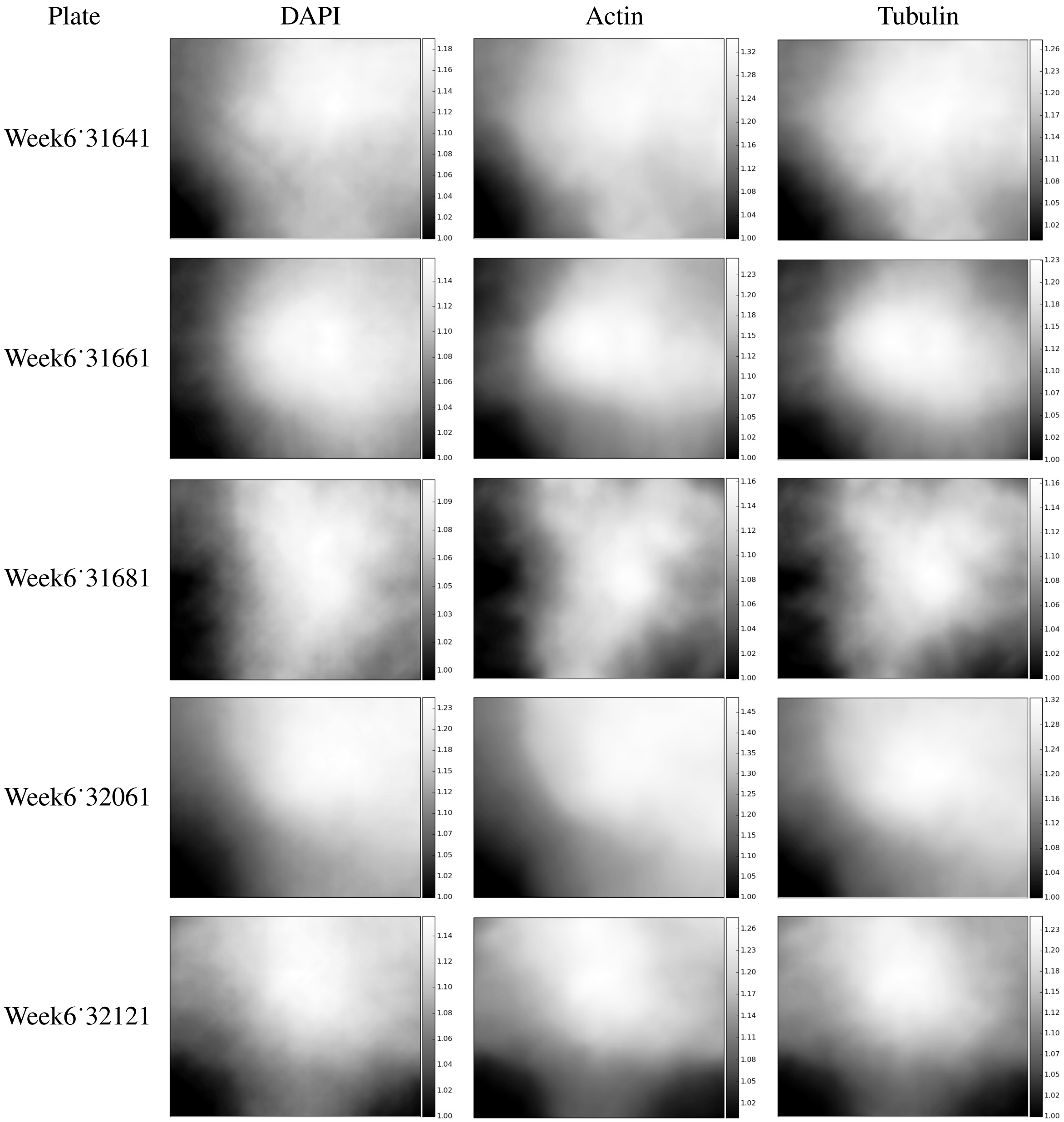

Figure S2: **Illumination correction functions based on plate-wise grouping (Plates 31-35).**

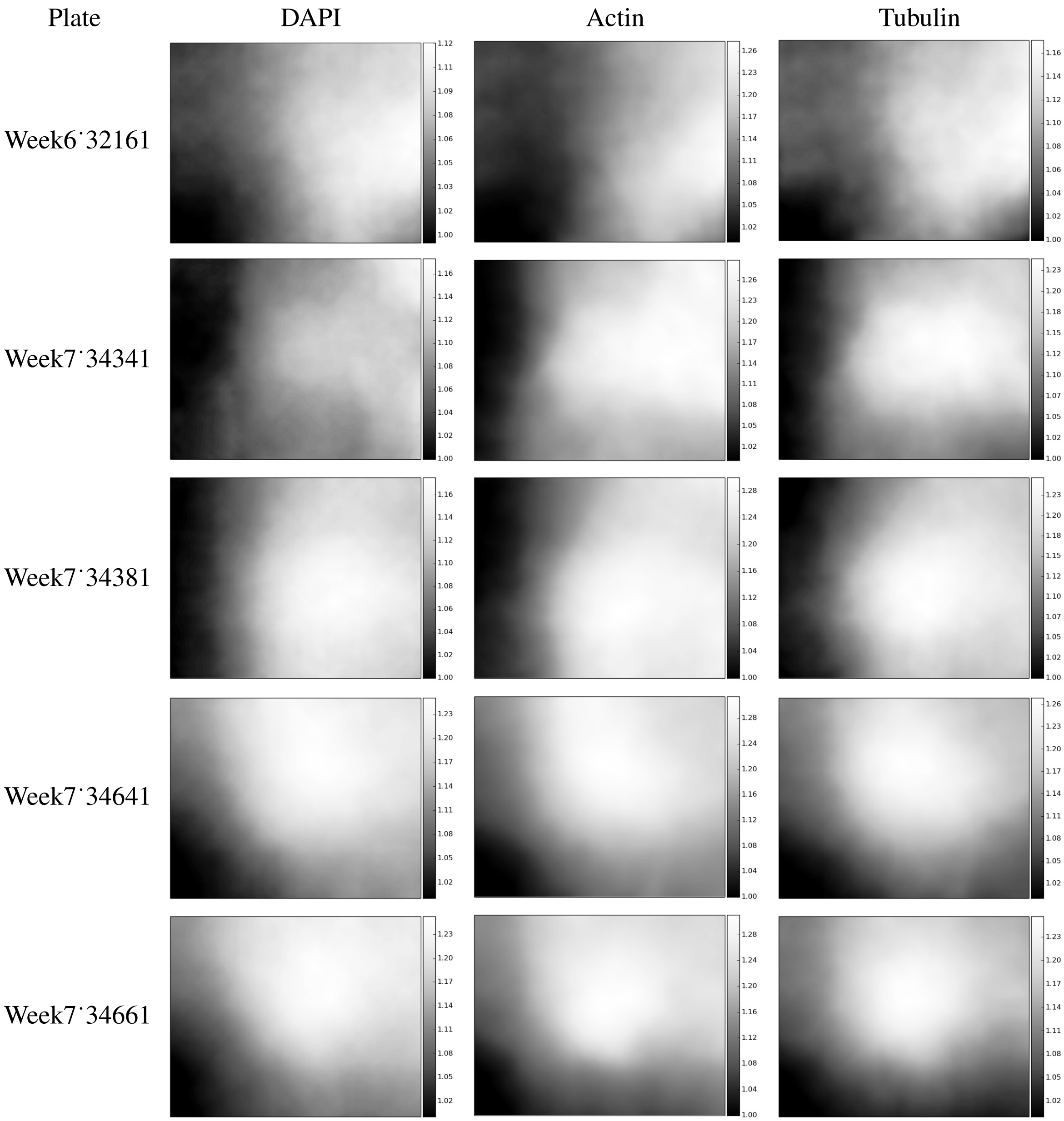

Figure S2: **Illumination correction functions based on plate-wise grouping (Plates 36-40).**

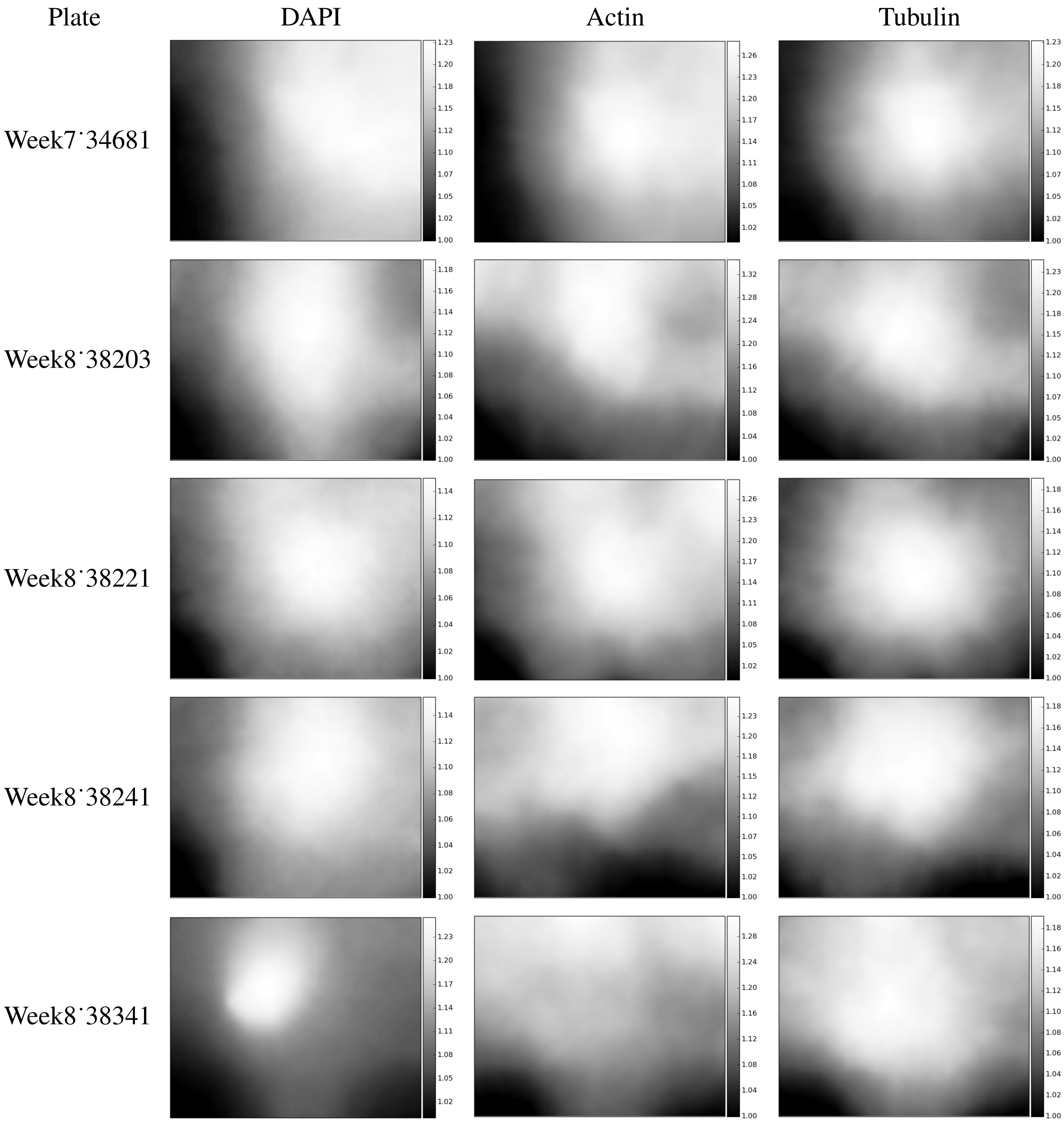

Figure S2: **Illumination correction functions based on plate-wise grouping (Plates 41-45).**

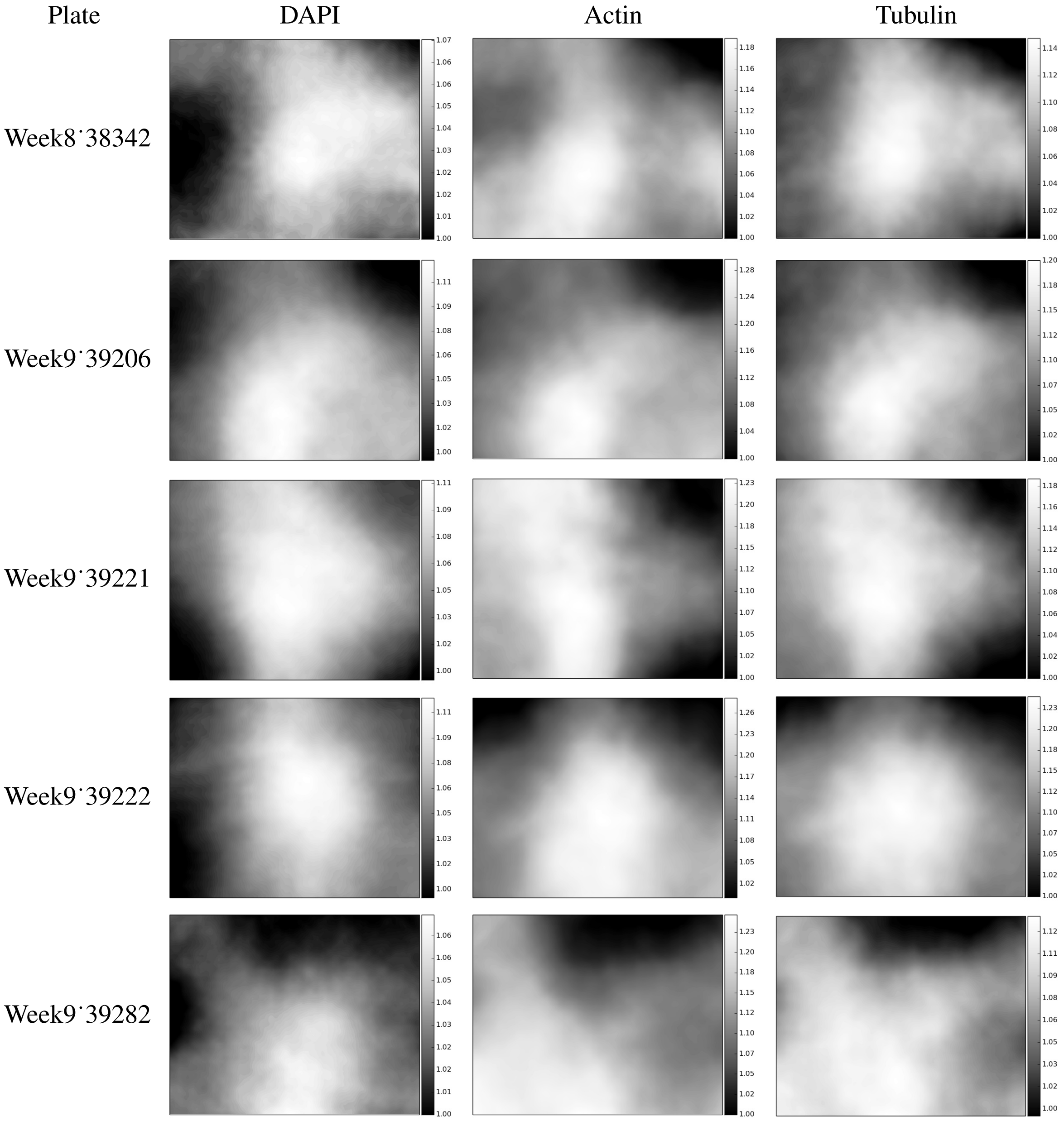

Figure S2: **Illumination correction functions based on plate-wise grouping (Plates 46-50).**

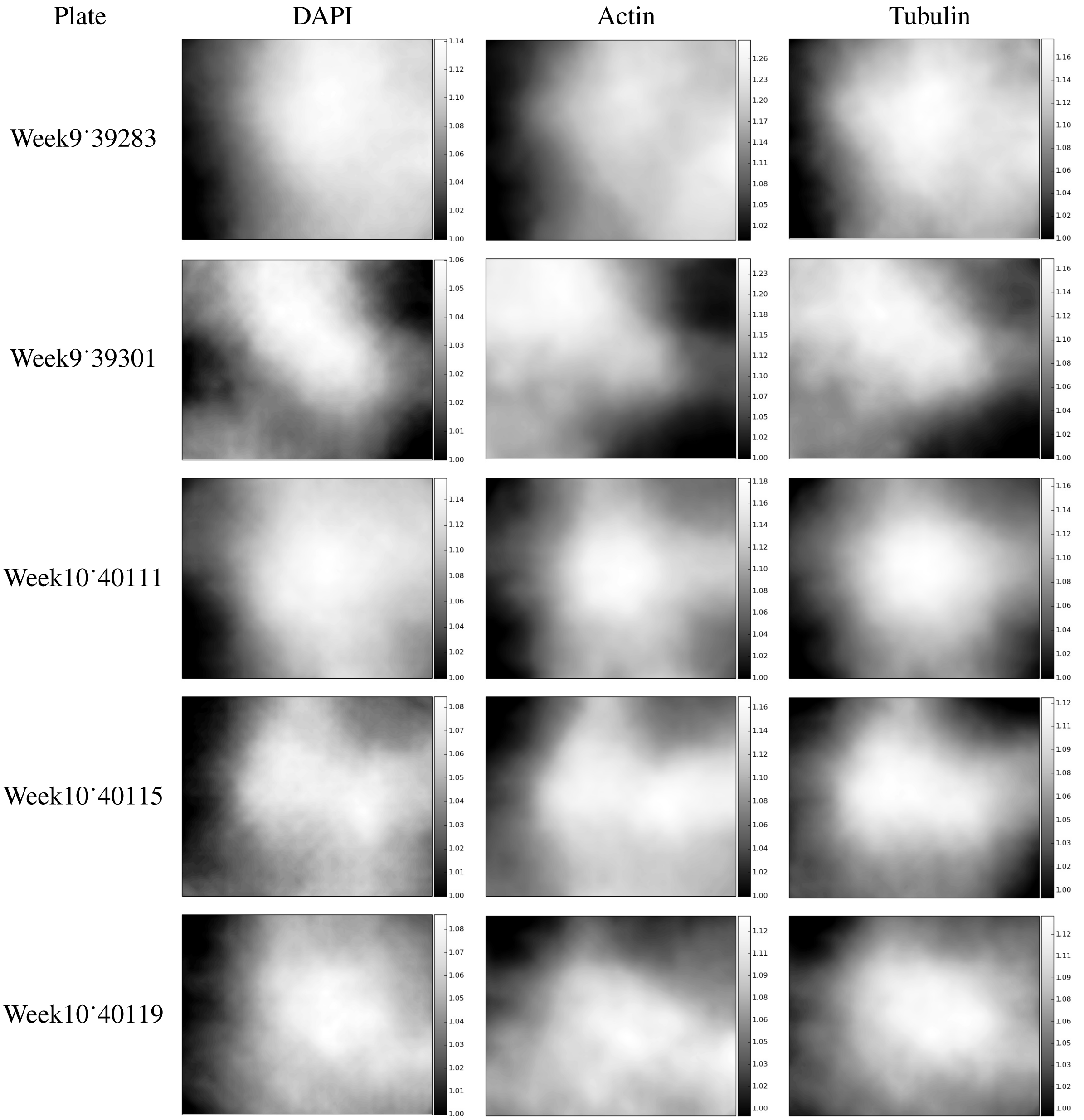

Figure S2: **Illumination correction functions based on plate-wise grouping (Plates 51-55).**

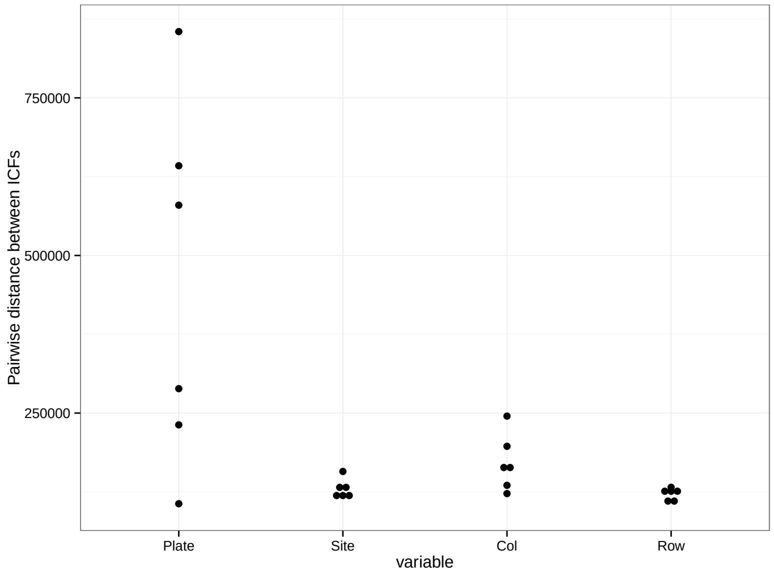

Figure S3: **Grouping of images to compute ICF.** Computing a single ICF for the entire experiment may result in a function that underfits the data, resulting in insufficient correction. Instead, ICFs can be computed for a given grouping of images, where images within a group are likely to have a common illumination function. In microtiter plate -based imaging experiments, there are four natural groupings of images - images from the same row, column, site or plate. Which grouping of images is optimal for computing an ICF? To test this, we computed 4 ICFs for each grouping, using 240 images per ICF, and measured the pairwise distance between the ICFs for each group. The plate-wise grouping results in distances that are significantly greater than all other groupings suggesting that grouping by plates will be most appropriate for this dataset. Note that the number of sites, or fields-of-view, in a typical HCS experiment varies from 4 to 16. The sites can correspond to arbitrary regions within a well, and are typically consistent across the whole experiment. In the BBBC021 data set, the sites have been ordered based on the order of acquisition.

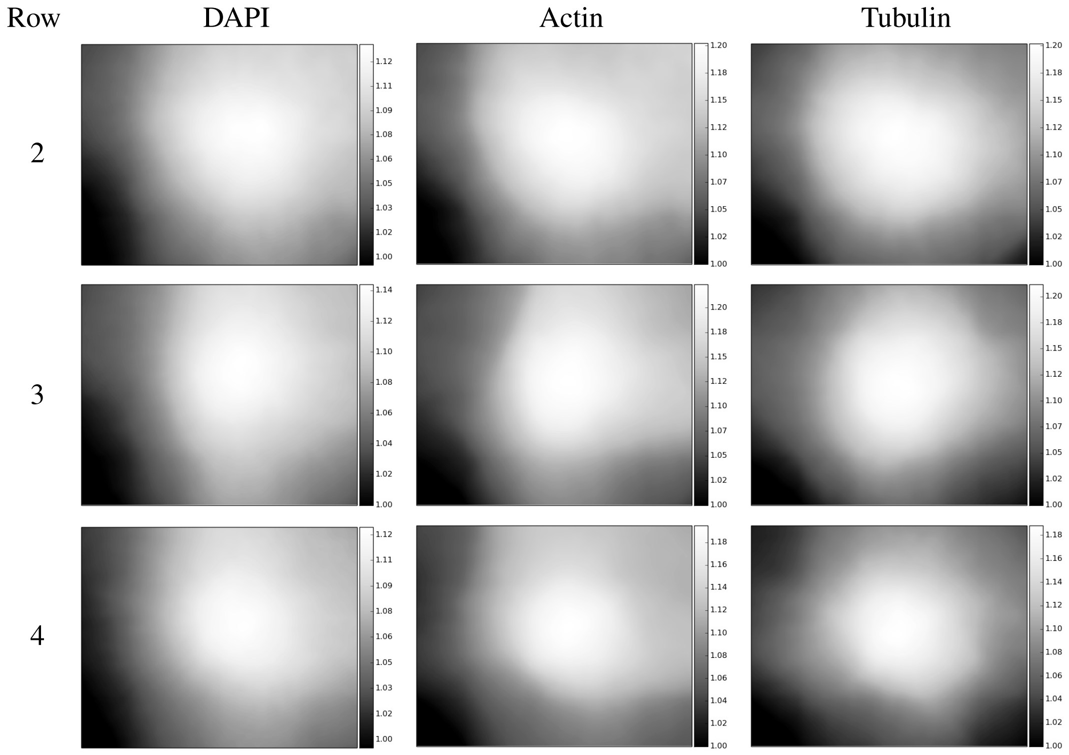

Figure S4: **Illumination correction functions based on row-wise grouping (Rows 2-4).**

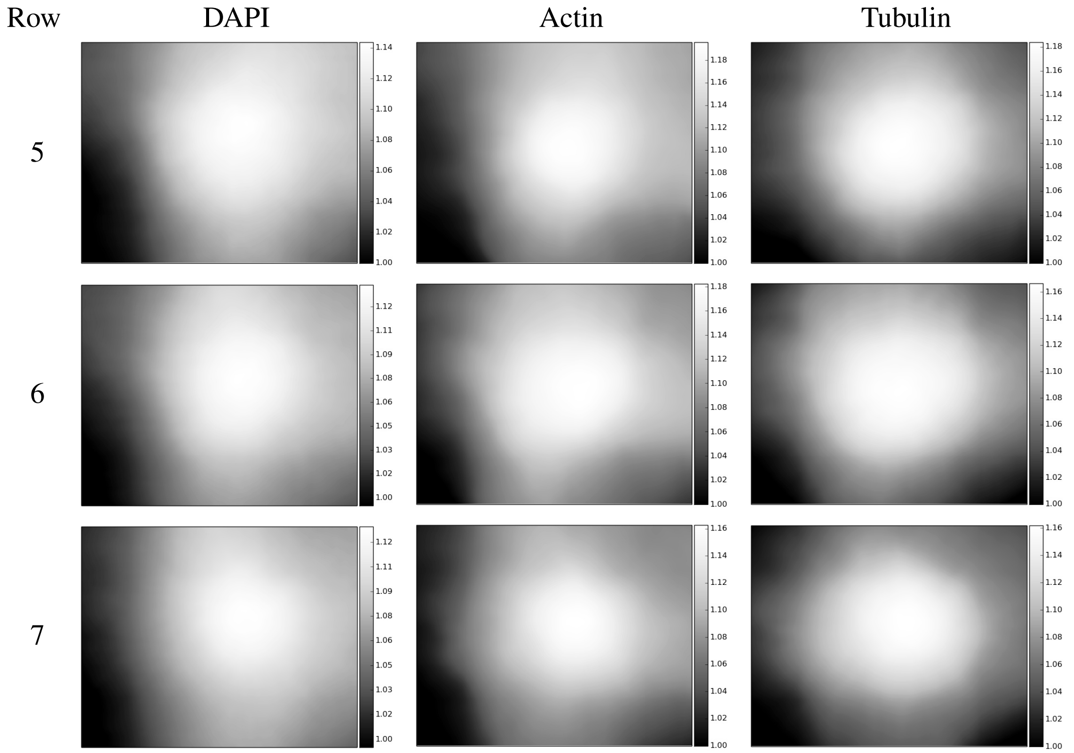

Figure S4: **Illumination correction functions based on row-wise grouping (Rows 5-7).**

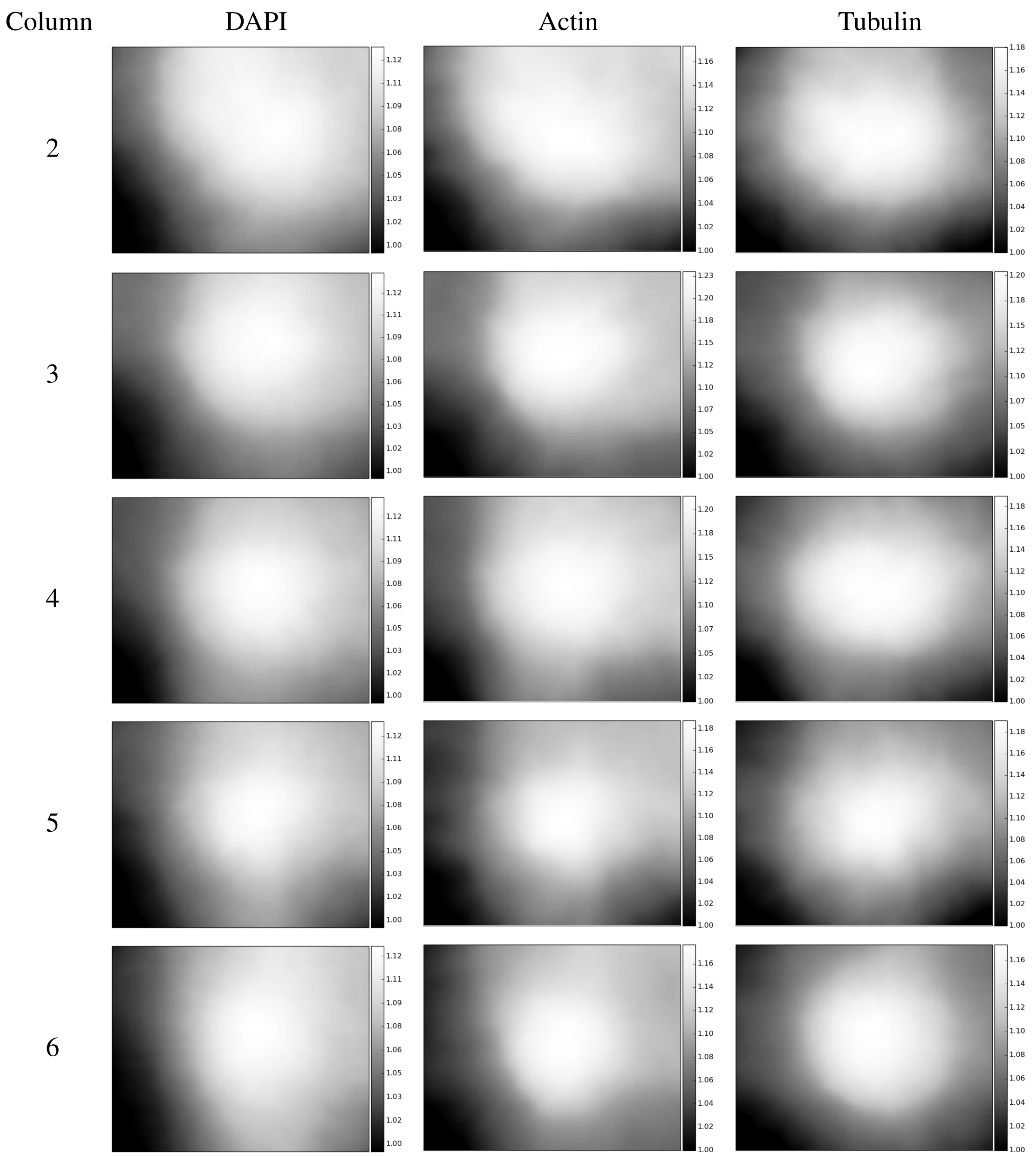

Figure S5: **Illumination correction functions based on column-wise grouping (Colums 2-6).**

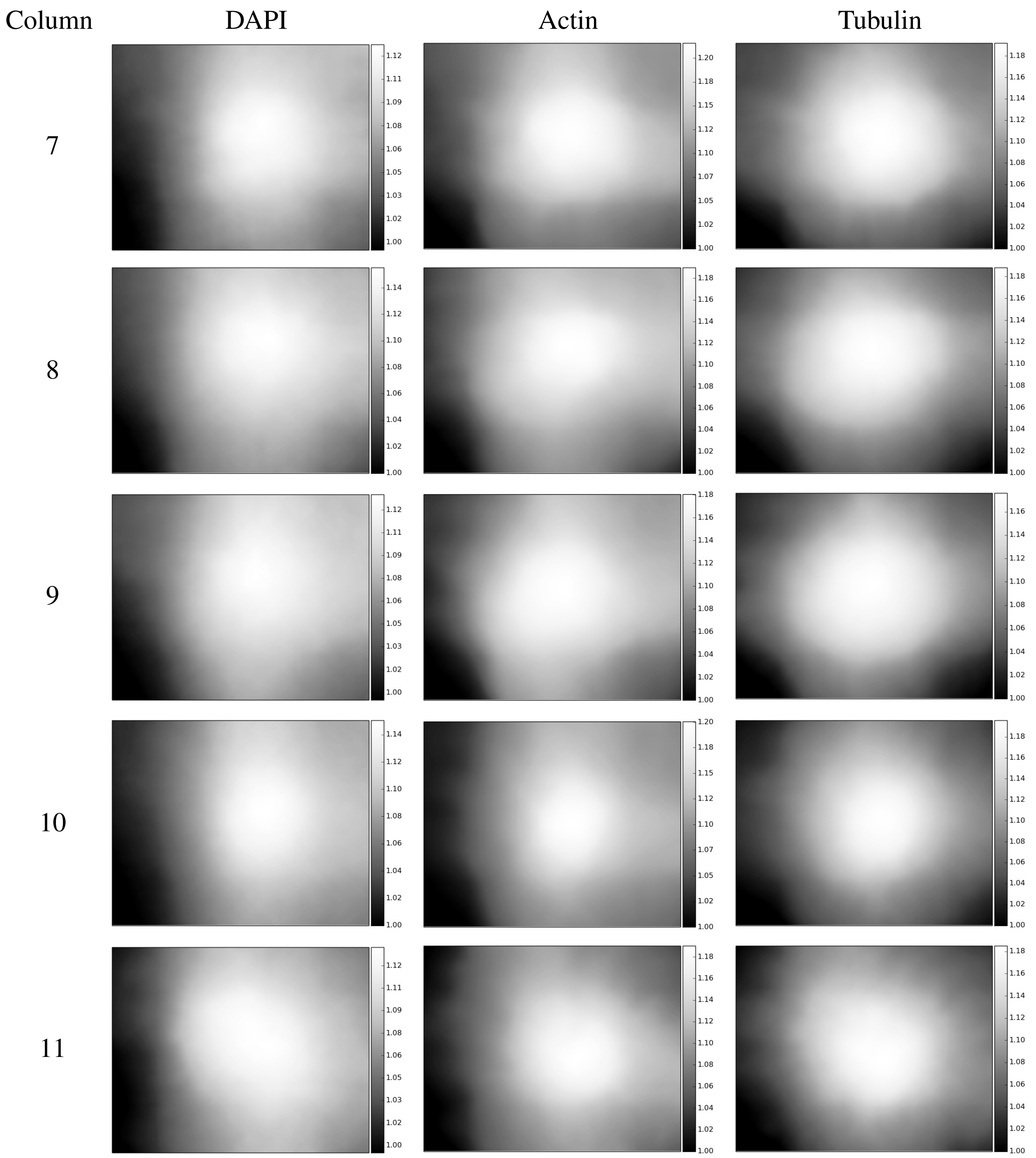

Figure S5: **Illumination correction functions based on column-wise grouping (Columns 7-11).**

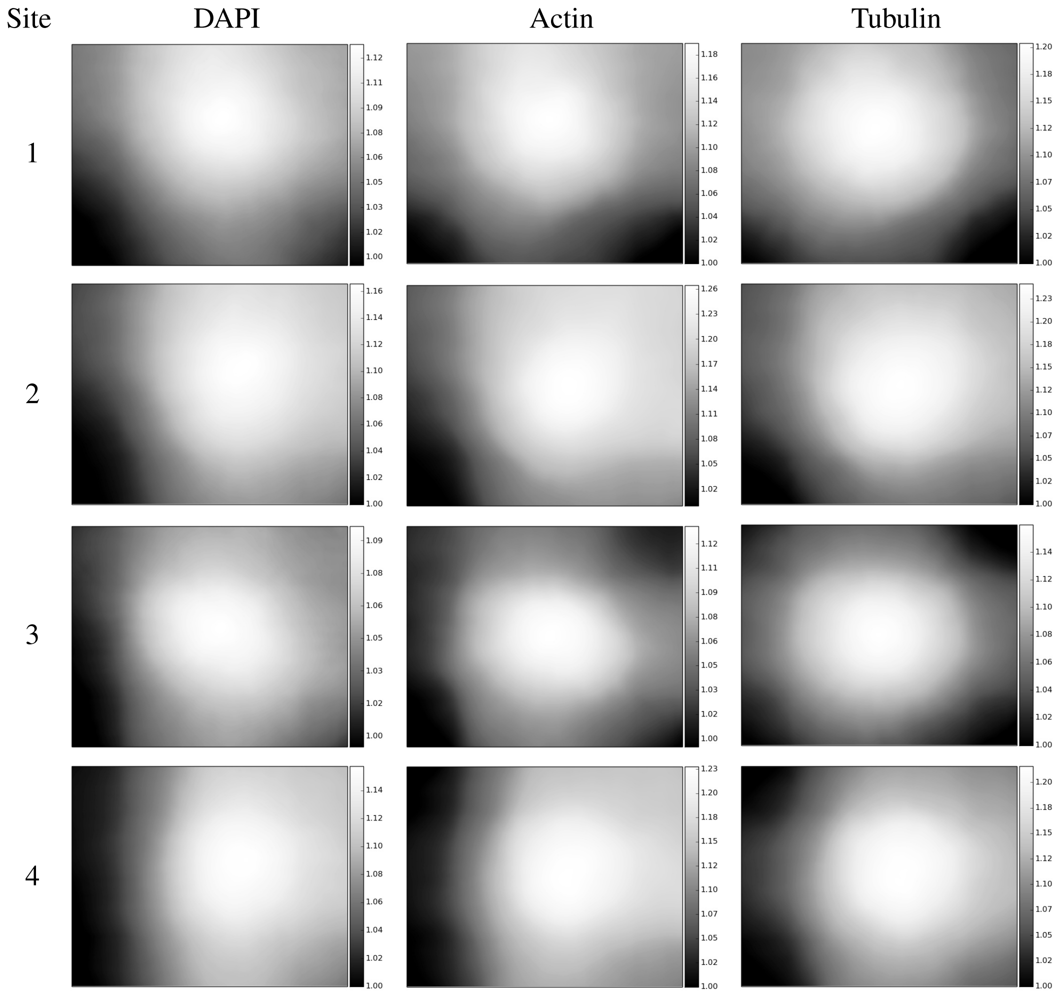

Figure S6: **Illumination correction functions based on site-wise grouping (Sites 1-4).**

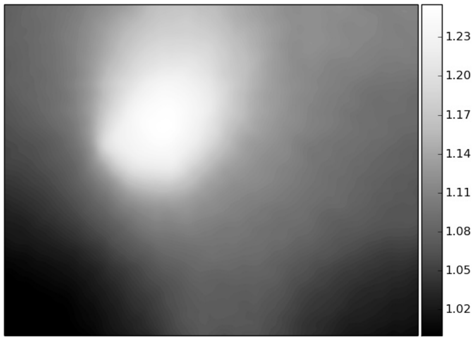

(a)

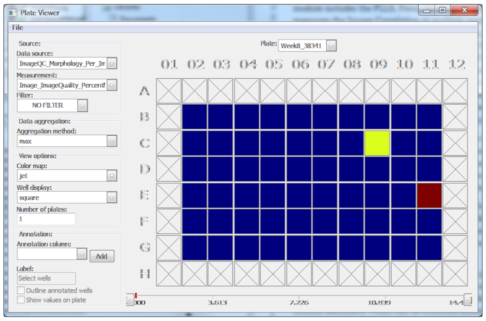

(b)

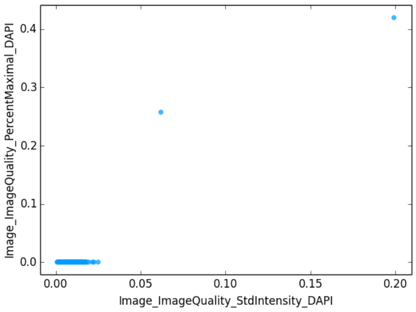

(c)

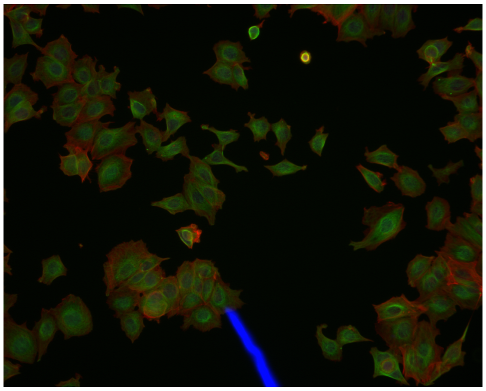

(d)

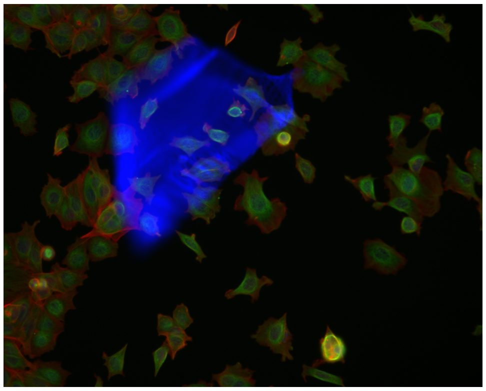

(e)

Figure S7: **Using ICFs for quality control**. (a) Visual inspection of Supporting Figure 2 revealed an anomalous ICF (Week8-38341, DAPI channel). (b) Based on the quality control best practices described in (Bray et al., 2012), the PercentMaximal feature (% of pixels that saturated) was visualized for the DAPI channel using the PlateViewer tool in CellProfiler Analyst. (c) The outliers can be clearly seen by plotting the standard deviation of the DAPI intensity versus the PercentMaximal of the DAPI channel using the ScatterPlot tool in CellProfiler Analyst. (d,e) The outlier wells C09 and E13 contain fluorescent artifacts (blue, mostly likely sample debris), producing the spatial aberration in the DAPI ICF for that plate.

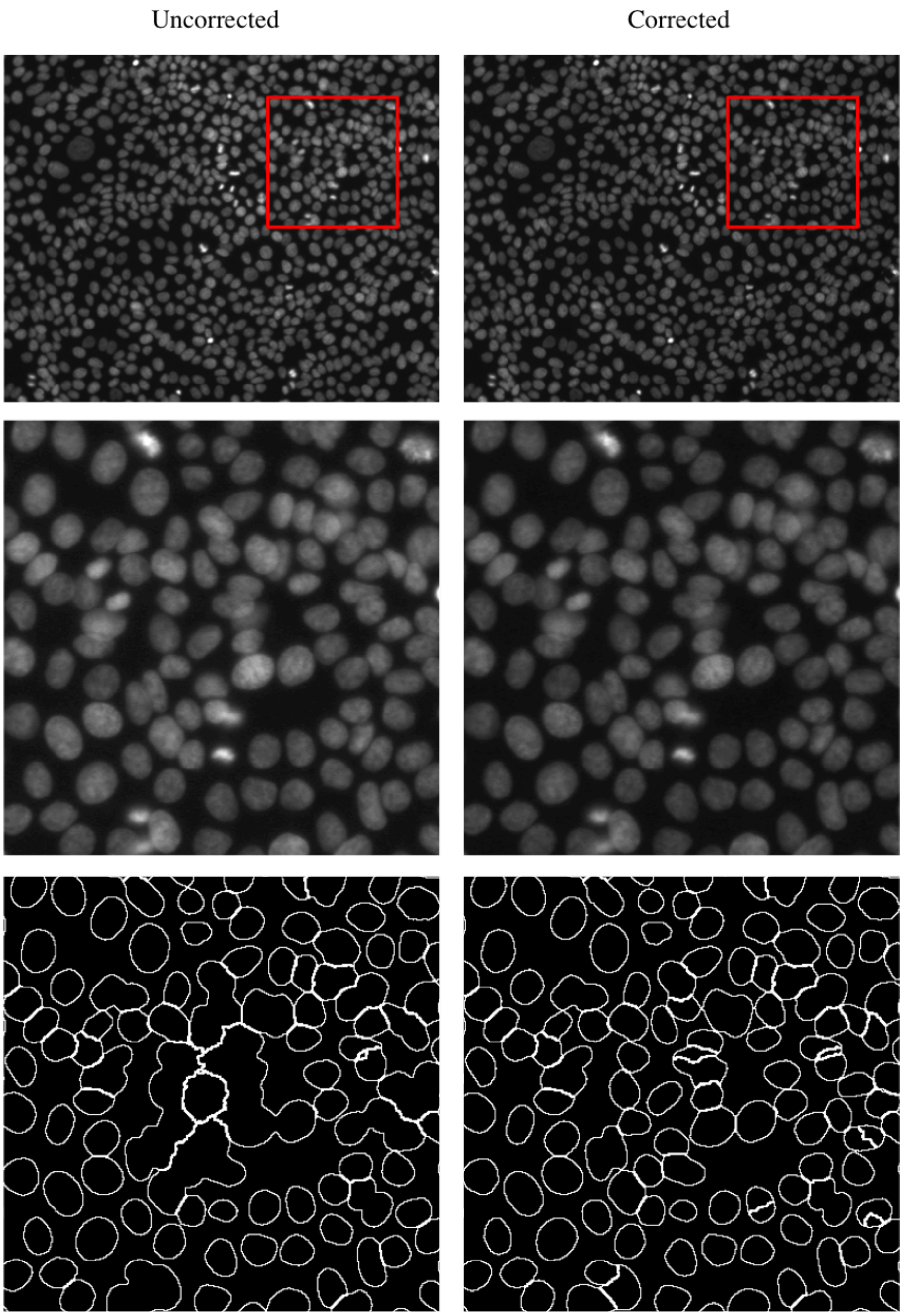

Figure S8: **Influence of illumination correction on segmentation quality.** This example shows a region of an image that demonstrates improvement in segmentation quality due to illumination correction. (Top row) DAPI channel image of the entire site, with the red square indicating the cropped region. (Middle row) Cropped region. (Bottom row) Outlines of cell nuclei. The cluster of cells in the center of the cropped image are better segmented after correcting for illumination inhomogeneity. The ICF used to correct this image is shown in Figure S2 (Week7˙34681, DAPI channel).

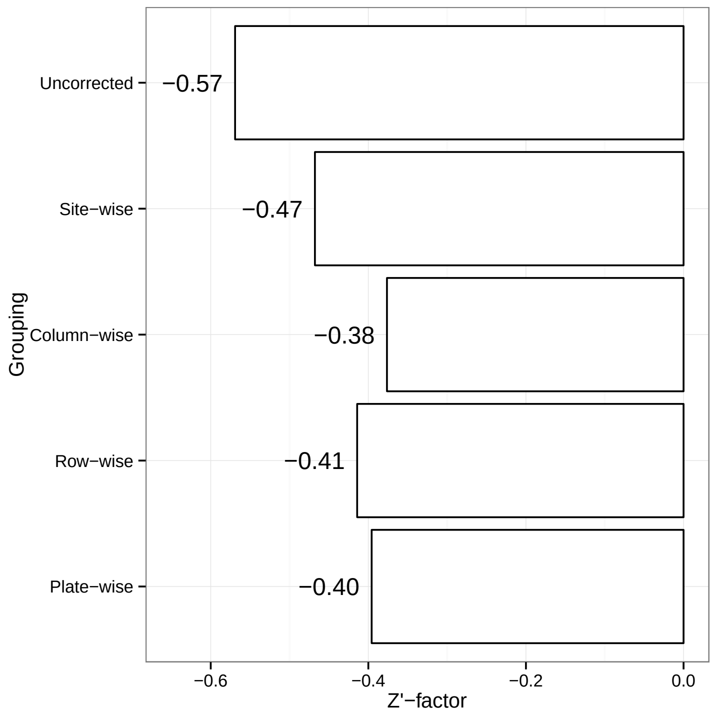

Figure S9: **Improvements in Z′-factor after illumination correction for a single readout.** Images were corrected for illumination using four different groupings as shown. The separation between positive and negative control for a single readout–total intensity of tubulin within each cell–was measured using Z′-factor. While column-wise grouping result in the greatest improvement in this experiment, plate-wise grouping results in improvement across many other features (Supporting Figure S10) and in our experience tends to be the most robust (see also Supporting S12).

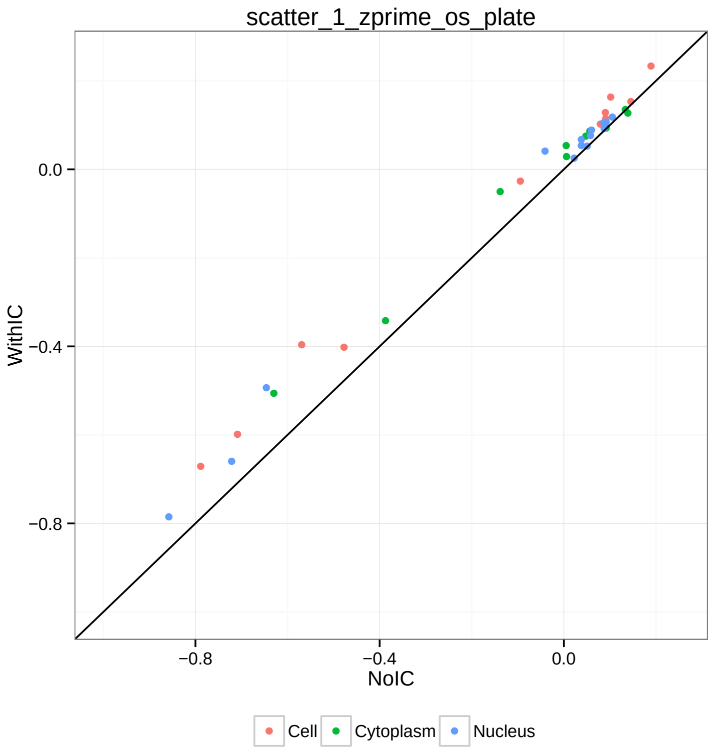

(a)

Figure S10: **Influence of illumination correction on assay quality - multiple features.** The analysis thus far has focused on a single feature – total intensity of tubulin in the cell. How much does the separation between positive and negative control change across other features? Here, we look at other Tubulin intensity features measured in the cell (red), cytoplasm (green), and nucleus (blue). The features include different percentiles of the tubulin intensity distribution in these compartments, as well as standard deviation, mean, and other statistics. In all, 42 features related to tubulin intensity are computed. Only those features with Z'-factor > -1 were retained for this analysis. The X- and Y- axes correspond to the Z'-factor value before and after correction respectively. As seen, the Z'-factor improves (increases) for nearly all the features. The ICFs used here correspond to a plate-wise grouping. The next three figures correspond to row-wise, column-wise, and site-wise grouping. The details of feature names as well as Z'-factor values corresponding to these figures is available in `Intensity_features_Zprime_improvement.csv` (Supporting Information).

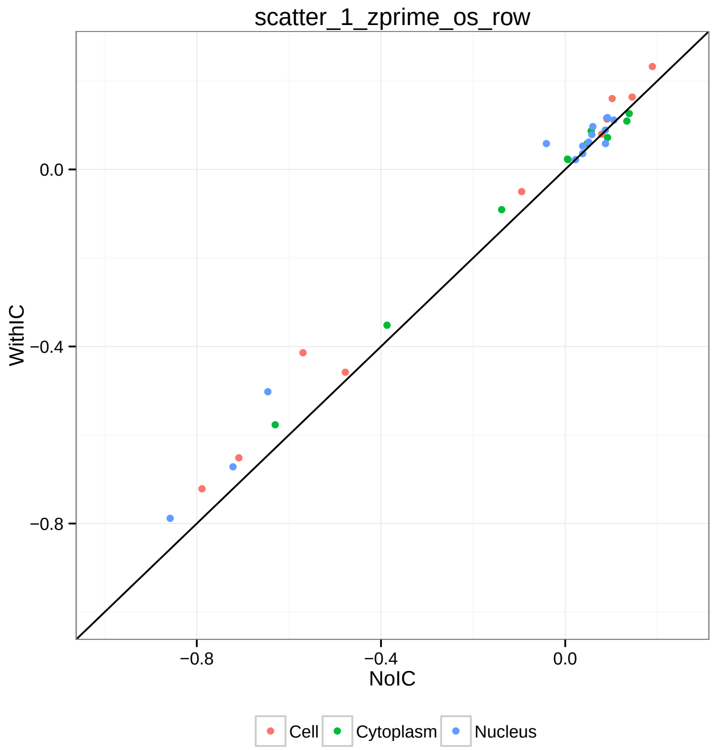

(b)

Figure S10: **Influence of illumination correction on assay quality - multiple features.** Same analysis as shown in Supporting Figure S10(a), but using row-wise grouping of images to compute ICFs.

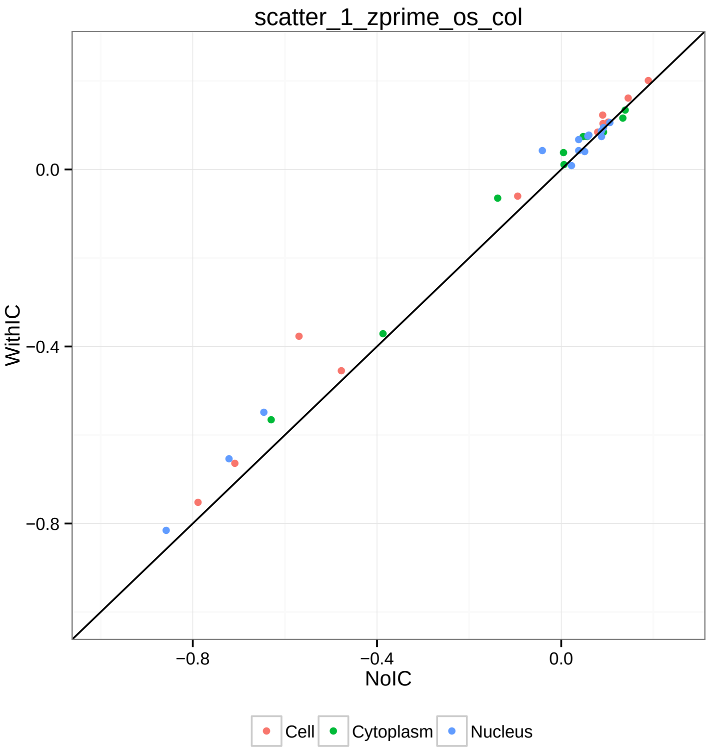

(c)

Figure S10: **Influence of illumination correction on assay quality - multiple features.** Same analysis as shown in Supporting Figure S10(a), but using column-wise grouping of images to compute ICFs.

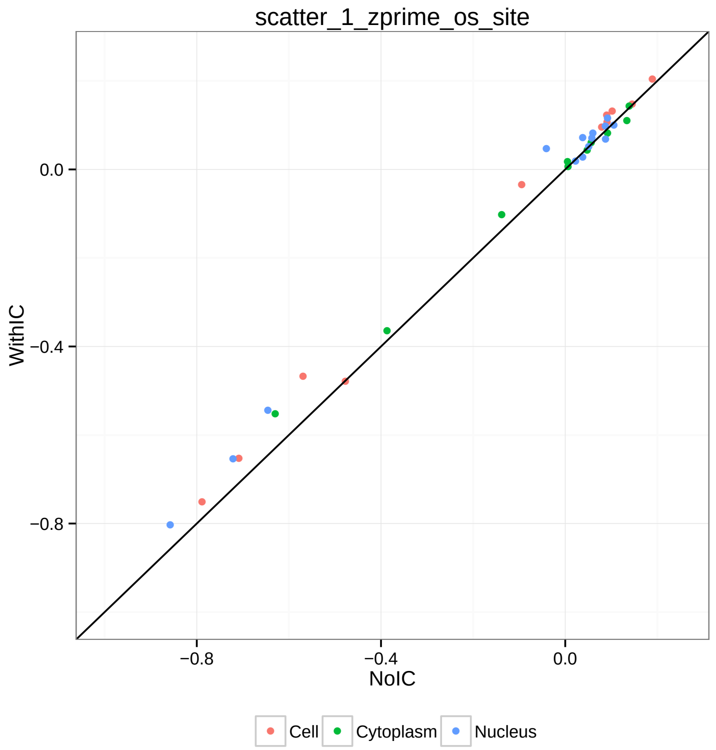

(d)

Figure S10: **Influence of illumination correction on assay quality - multiple features.** Same analysis as shown in Supporting Figure S10(a), but using site-wise grouping of images to compute ICFs.

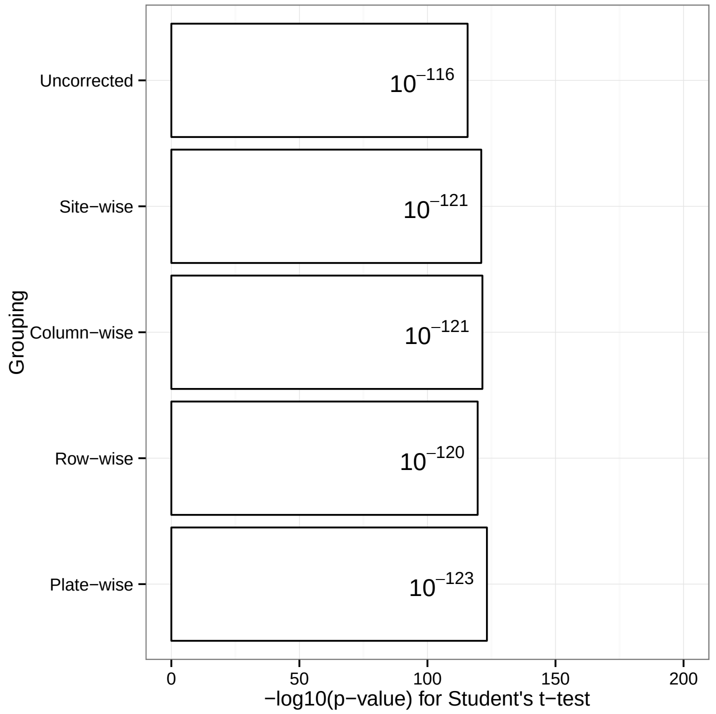

Figure S11: **Improvements in separation between means after illumination correction for a single readout.** Same analysis as Figure S9, but testing for separation between means instead of Z′-factor. The separation between control means is reported on the x-axis using $-log10(p)$, where p is the p-value from the Student t-test. The actual p-values for each grouping is also shown. Plate-wise grouping results in the greatest increase in significance.

Row-wise ICF

True mechanistic class		Act	Aur	Ch	DD	DR	Eg5	Epi	KI	MD	MS	PD	PS	Acc.
Actin disruptors	Act	1						1					3	20 %
Aurora kinase inhibitors	Aur		12											100 %
Cholesterol-lowering	Ch			4				2						67 %
DNA damage	DD				9									100 %
DNA replication	DR					8								100 %
Eg5 inhibitors	Eg5						12							100 %
Epithelial	Epi			1				7						88 %
Kinase inhibitors	KI								5					100 %
Microtubule destabilizers	MD				1		1			12				86 %
Microtubule stabilizers	MS						1				8			89 %
Protein degradation	PD											7		100 %
Protein synthesis	PS												8	100 %

Predicted class. Overall accuracy: 93 / 103 = **90 %**

Column-wise ICF

True mechanistic class		Act	Aur	Ch	DD	DR	Eg5	Epi	KI	MD	MS	PD	PS	Acc.
Actin disruptors	Act	1						1					3	20 %
Aurora kinase inhibitors	Aur		12											100 %
Cholesterol-lowering	Ch	1		2			1	2						33 %
DNA damage	DD				9									100 %
DNA replication	DR					8								100 %
Eg5 inhibitors	Eg5						12							100 %
Epithelial	Epi							7	1					88 %
Kinase inhibitors	KI								5					100 %
Microtubule destabilizers	MD				1		1			12				86 %
Microtubule stabilizers	MS						1				8			89 %
Protein degradation	PD											7		100 %
Protein synthesis	PS												8	100 %

Predicted class. Overall accuracy: 91 / 103 = **88 %**

Site-wise ICF

True mechanistic class		Act	Aur	Ch	DD	DR	Eg5	Epi	KI	MD	MS	PD	PS	Acc.
Actin disruptors	Act	1						1					3	20 %
Aurora kinase inhibitors	Aur		12											100 %
Cholesterol-lowering	Ch			3				2					1	50 %
DNA damage	DD				9									100 %
DNA replication	DR					8								100 %
Eg5 inhibitors	Eg5						11				1			92 %
Epithelial	Epi							7	1					88 %
Kinase inhibitors	KI								5					100 %
Microtubule destabilizers	MD				1		3			10				71 %
Microtubule stabilizers	MS						1				8			89 %
Protein degradation	PD											7		100 %
Protein synthesis	PS												8	100 %

Predicted class. Overall accuracy: 89 / 103 = **86 %**

Figure S12: **Influence of illumination correction on image-based profiling**. As seen in Figure 1 where ICFs were computed by plate-wise grouping of images, illumination correction improves accuracy of mechanism-of-action classification of compounds by 6%. Here, confusion matrices are shown for row-, column-, and site-wise grouping of images.
