# Supplementary material for: Pipeline for illumination correction of images for high-throughput microscopy
Source: J Microsc. 2014 Sep 16;256(3):231–6. doi: 10.1111/jmi.12178 (PMC4359755; doi:10.1111/jmi.12178)
Supplement: Appendix — Source Code & Definitions. [file jmi0256-0231-SD2.docx]

Supporting Information

## Definitions

### One-tailed Z'-factor

This measure is an attempt to be more robust against skewed population distributions. This statistic is computed similar to the Z'-factor but using only those samples that lie between the positive and negative control population means to calculate the standard deviations:


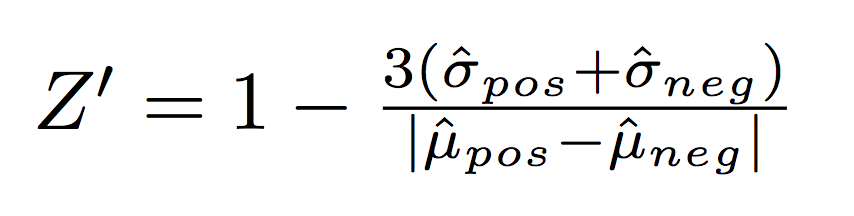


where
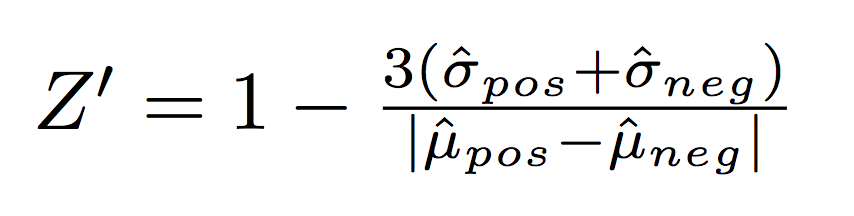
and
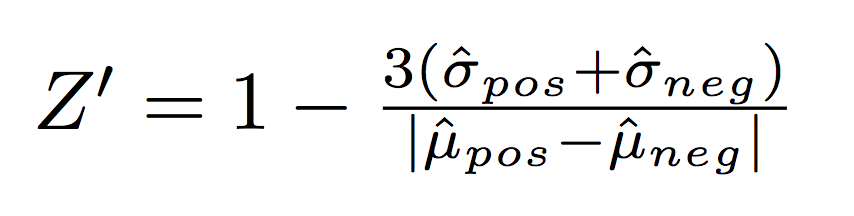
 correspond to the standard deviations of the positive and negatives controls, estimated using the modification described above. The statistic has been implemented in the accompanying source code (analysis_univariate/code/analysis.R). See (Bray and Carpenter, 2013) for further details on rationale, interpretation, advantages and disadvantages.

## Software

The image analysis pipelines can be loaded and run using CellProfiler 2.1.0 (revision 0c7fb94) available at <http://www.cellprofiler.org>.

The data analysis scripts require the following software

- CellProfiler Analyst (<http://github.com/CellProﬁler/CellProﬁler-Analyst/>). Clone repository using git clone <https://github.com/CellProfiler/CellProfiler-Analyst.git>. Add the CellProfiler-Analyst folder to the PYTHONPATH variable.
- R 2.15. Missing R libraries can be installed on the R prompt using install.packages. E.g. install.packages(“plyr”)
- Python 2.7.2. Unless otherwise noted, all python dependencies are available through the Python Package Index (PyPi, <http://pypi.python.org/pypi/>)

## Image data sets

### The high-throughput image set BBBC021v1 (Caie *et al.*, 2010) from the Broad Bioimage Benchmark Collection (Ljosa *et al.*, 2012) was used for all the experiments in this paper. The data set is freely available for download at <http://www.broadinstitute.org/bbbc/BBBC021/>.

### Organize the data as follows. Create a folder names MorphologyAssay and then 10 sub-folders named Week1, Week2, …, Week10. Download the zip files from the link above and move them into their corresponding Week folders, e.g., move BBBC021_v1_images_Week1_22123.zip into Week1. Unzip the files. Finally, edit the path information in the CSV files to point to the appropriate location. E.g. In the CSV files, change PARENT_FOLDER/MorphologyAssay to the appropriate location.

###

##

## Pipelines and scripts

Archive containing all files described is available on the website below:
[http://www.broadinstitute.org/pubs/singh_jmicroscopy_2014/](http://www.broadinstitute.org/pubs/singh_jmicroscopy_2014/)

### Illumination correction

(Folder: pipelines/)

Illum_Median500M.cppipe implements the illumination correction pipeline. The LoadData module of this pipeline requires the CSV file supplement_allwells_in_plate_Filelist_Metadata_unix.csv.

Illum_Median500M.cppipe has been implemented for plate-wise grouping of images. To change this to row-, column-, or site-wise, select the corresponding option in “Select Metadata tags for grouping” in the LoadData module. The options that appear in this box are simply the fields in the CSV file that have a prefix of “Metadata_”.

**Image analysis**

(Folder: pipelines/)

Analysis_posneg_withoutICFs.cppipe and Analysis_posneg_withICFs_Median500M.cppipe

implement the image analysis pipelines to generate the readouts used in Section “Influence of illumination correction on assay quality: screens using a univariate readout”. The LoadData module of these pipelines requires the CSV file posneg_Filelist_trimmed_row_col_site.csv.

Analysis_posneg_withoutICFs.cppipe does not use ICFs and therefore corresponds to the “without correction” case.

Analysis_posneg_withICFs_Median500M.cppipe uses previously computed ICFs and corresponds to the “with correction” case . The field “Input image file location” in the LoadSingleImage module should be updated to point to the output folder that is created after running Illum_Median500M.cppipe. The settings in the LoadSingleImage module correspond to ICFs computed using plate-wise grouping. This can be changed to other groupings by appropriately editing the field “Filename of the image to load” (i.e. change “Plate” to “Row”, “Site”, or “Col” by right-clicking on the text and select the appropriate tag).

To generate the readouts used in Section “Influence of illumination correction on assay quality: experiments involving multivariate profiling”, the two pipelines need to be modified slightly as follows. In the LoadData module, the CSV file to be loaded should be changed to supplement_Filelist_Metadata_row_col_site_unix.csv. In the ExportToDatabase module, the field “Table Prefix” should be changed to point to a new table (e.g. in Analysis_posneg_withoutICFs.cppipe, change MorphologyAnalysis_posneg_WithoutICFs_ to MorphologyAnalysis_WithoutICFs_)

#### Description of the image analysis pipeline

For a detailed description of each step of the image analysis pipeline, load the cppipe files CellProfiler and inspect each module, clicking on the “?” button for an explanation of each parameter. Below, we provide a brief summary of the pipeline.

Images are loaded, and in the case of “withICFs” pipelines, illumination correction is performed by multiple each channel by the corresponding ICF (See the first three ImageMath modules, corresponding to the three channels).

Nuclei are then identified in the IdentifyPrimaryObjects module as follows. Two-class Otsu thresholding is used to identify the foreground in the DAPI channel, and objects outside a predefined diameter range are discarded, as well as those touching the border of the image. Cells are declumped using object shape as the parameter. Parameter settings for the declumping can be found in the pipeline file. Finally, holes in the identified objects are filled.

Next, cells outlines are obtained using the IdentifySecondaryObjects module. Two-class Otsu thresholding is used to identify the foreground in the Actin channel. Then, the nuclei identified in the DAPI channel are used to “seed” the detection of cell outlines in the Actin channel using the propagation technique (for details, click on “?” next to the option “Select the method to identify the secondary objects” in the IdentifySecondaryObjects module). The cytoplasm is identified using the IdentifyTertiaryObjects module by removing the identified nuclear region from the identified cellular region.

Finally, the “Measure” modules are used to extract features from the images, using the segmentations obtained above as masks.

**Data analysis**

The instructions below are for executing the commands in a POSIX terminal. It assumes that the folder structure created on unzipping reproduce.zip in maintained.

#### Influence of illumination correction on assay quality: screens using a univariate readout

(Folder: analysis_univariate/)

1. Get the appropriate version of CellProfiler Analyst

# cd to the CellProfiler-Analyst folder

git checkout 3adc45b

2. Create summaries of per-cell readouts.

cd analysis_univariate/code

DATASET=AZ_posneg_WithICFs_Median500M

# Create local cache

./1-create_cache.py $DATASET

# Normalize data

./2-create_normalizations.py $DATASET

# Create per-well summaries of data

./3-create_profiles.sh $DATASET

The above generates summaries for readouts corresponding plate-wise grouping. For the uncorrected dataset, and for row-, column-, and site-wise grouping datasets repeat the above after changing DATASET variable appropriately as below:

DATASET=AZ_posneg_WithoutICFs

DATASET=AZ_posneg_WithICFs_Median500M_row

DATASET=AZ_posneg_WithICFs_Median500M_col

DATASET=AZ_posneg_WithICFs_Median500M_site

3. Compute Z’-factor values and related plots.

Rscript analysis.R

Note: The details of feature names as well as Z’-factor values corresponding to Figures S9(a-d) is generated in Intensity_features_Zprime_improvement.csv. Additional information about the intensity features is available in the online CellProfiler documentation at <http://www.cellprofiler.org/CPmanual/MeasureObjectIntensity.html>

#### Influence of illumination correction on assay quality: experiments involving multivariate profiling

(Folder: analysis_multivariate/)

1. Get the appropriate version of CellProfiler Analyst

# cd to the CellProfiler-Analyst folder

git checkout pmid_24045582

2. Create cache, per-well summaries of data and compute confusion matrix

# create cache

DATASET=AZ_Median500M

cd analysis_univariate/$DATASET/src

python -m cpa.profiling.cache -r \

../properties/supplement.properties \

../supplement_cache/ "compound = 'DMSO'"

# create per-well summaries of data and compute confusion matrix

make ../figures/confusion_a.pdf

The above generates the confusion matrix corresponding plate-wise grouping. For the uncorrected dataset, and for row-, column-, and site-wise grouping datasets repeat the above after changing DATASET variable appropriately as below:

DATASET=AZ_WithoutICFs

DATASET=AZ_WithICFs_Median500M_row

DATASET=AZ_WithICFs_Median500M_col

DATASET=AZ_WithICFs_Median500M_site
